# Short‐term protein restriction at advanced age stimulates FGF21 signalling, energy expenditure and browning of white adipose tissue

**DOI:** 10.1111/febs.15604

**Published:** 2020-11-09

**Authors:** Marleen B. Dommerholt, Maaike Blankestijn, Marcel A. Vieira‐Lara, Theo H. van Dijk, Henk Wolters, Mirjam H. Koster, Albert Gerding, Ronald P. van Os, Vincent W. Bloks, Barbara M. Bakker, Janine K. Kruit, Johan W. Jonker

**Affiliations:** ^1^ Sections of Molecular Metabolism and Nutrition Department of Pediatrics University Medical Center Groningen University of Groningen Groningen the Netherlands; ^2^ Sections of Systems Medicine of Metabolism and Signaling Department of Pediatrics University Medical Center Groningen University of Groningen Groningen the Netherlands; ^3^ Department of Laboratory Medicine University Medical Center Groningen University of Groningen the Netherlands; ^4^ Mouse Clinic for Cancer and Aging Central Animal Facility University Medical Center Groningen University of Groningen Groningen the Netherlands

**Keywords:** browning of white adipose tissue, dietary protein restriction, energy metabolism, FGF21 signalling, thermogenesis

## Abstract

Dietary protein restriction has been demonstrated to improve metabolic health under various conditions. However, the relevance of ageing and age‐related decline in metabolic flexibility on the effects of dietary protein restriction has not been addressed. Therefore, we investigated the effect of short‐term dietary protein restriction on metabolic health in young and aged mice. Young adult (3 months old) and aged (18 months old) C57Bl/6J mice were subjected to a 3‐month dietary protein restriction. Outcome parameters included fibroblast growth factor 21 (FGF21) levels, muscle strength, glucose tolerance, energy expenditure (EE) and transcriptomics of brown and white adipose tissue (WAT). Here, we report that a low‐protein diet had beneficial effects in aged mice by reducing some aspects of age‐related metabolic decline. These effects were characterized by increased plasma levels of FGF21, browning of subcutaneous WAT, increased body temperature and EE, while no changes were observed in glucose homeostasis and insulin sensitivity. Moreover, the low‐protein diet used in this study was well‐tolerated in aged mice indicated by the absence of adverse effects on body weight, locomotor activity and muscle performance. In conclusion, our study demonstrates that a short‐term reduction in dietary protein intake can impact age‐related metabolic health alongside increased FGF21 signalling, without negatively affecting muscle function. These findings highlight the potential of protein restriction as a strategy to induce EE and browning of WAT in aged individuals.

## Graphical abstract

A reduced dietary protein intake has been demonstrated to improve metabolic health under various conditions. Here, it is shown that a short‐term 7% low‐protein dietary intervention can impact age‐related metabolic health in 18‐month‐old animals. Alongside increased plasma fibroblast growth factor 21 levels, a low‐protein diet induces energy expenditure and browning of white adipose tissue in 18‐month‐old mice, without negatively affecting muscle strength.

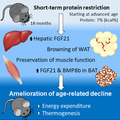

**Abbreviations**
BAT: brown adipose tissue
BCAA: branched‐chain amino acid
CR: caloric restriction
DEG: differentially expressed gene
EE: energy expenditure
FGF21: fibroblast growth factor 21
H&E: haematoxylin and eosin
HP: high protein
LP: low protein
MP: medium protein
NEFA: nonesterified fatty acid
RER: respiratory exchange ratio
T2D: type 2 diabetes mellitus
UCP1: uncoupling protein 1
WAT: white adipose tissue

## Introduction

Ageing and age‐associated changes in lifestyle are known to affect a wide range of metabolic processes. Ageing itself is one of the main risk factors for the development of chronic metabolic diseases, including type 2 diabetes (T2D), cardiovascular disease and cancer [1, 2, 3]. With the worldwide increase in life expectancy and median lifespan over the last few decades, understanding the mechanisms by which ageing affects metabolic processes has become an increasingly important research focus [4, 5]. Caloric restriction (CR) is one of the most effective strategies to delay the symptoms of ageing and to extend longevity in a wide variety of animals [6]. CR, however, is difficult to maintain, and its long‐term success has been limited by the poor adherence to this diet. Recently, changing macronutrient balance has emerged as a more feasible alternative for CR [7, 8, 9, 10]. Long‐term dietary protein restriction without reducing total caloric intake has been shown to have similar beneficial effects on metabolic health and extension of longevity in mice as CR [11]. Within 1 week after the dietary switch, mice were protected against obesity and resistant to cold stress as a result of increased energy expenditure (EE) and increased utilization of free fatty acids and glucose in brown adipose tissue (BAT) [12, 13, 14, 15].

Mechanisms underlying the beneficial metabolic effects of protein restriction have been associated with multiple pathways including mTOR and fibroblast growth factor 21 (FGF21) signalling. FGF21 has recently emerged as an endocrine signal associated with metabolic control, as it is increased in response to fasting, starvation, protein restriction and physical exercise as well as overfeeding, ageing and metabolic diseases such as obesity, T2D and nonalcoholic fatty liver disease [16, 17, 18, 19]. FGF21 signalling is associated with improved metabolic health and longevity as transgenic mice overexpressing FGF21 exhibit increased lifespan and share a number of beneficial phenotypes with long lived dwarf mice [20]. Currently, clinical trials are approved for FGF21 analogues in T2D patients to lower body weight, insulin levels and plasma triglycerols (TG) [21]. In response to a low‐protein (LP) diet, transcription of *Fgf21* is rapidly induced in the liver by both PPARα signalling and the GCN2/eIF2 pathway, and FGF21 is subsequently secreted into the circulation [13, 22]. Work on (hepatic) FGF21‐deficient mice showed that the LP diet‐induced effects on basal‐ and cold‐induced EE, insulin sensitivity, glucose metabolism and fatty acid clearance were FGF21‐dependent [12, 23, 24, 25]. Together, these studies suggest that protein restriction induced hepatic FGF21 production and activated BAT and white adipose tissue (WAT) to increase thermogenesis, resulting in increased EE [22, 26, 27].

Ageing is, however, associated with diminished lipid handling, defective thermogenesis and impaired *de novo* adipogenesis in subcutaneous WAT and BAT, which together contribute to the development of age‐dependent insulin resistance and dyslipidaemia [28, 29, 30]. During ageing, the expansion of subcutaneous WAT is accompanied by a decrease in thermogenic capacity of this depot, reflected by decreased expression of the key thermogenic marker uncoupling protein 1 (*Ucp1*) [29] and a reduced ability to maintain constant body temperature in response to cold exposure [30]. Whether a short‐term protein restriction, starting at an advanced age, has beneficial effects on metabolic health is not known. Here, we asked whether a short‐term dietary intervention with reduced protein content would be able to improve metabolic health at an advanced age. Using aged mice, we examined the effects of an LP diet on FGF21 levels, insulin sensitivity, EE and thermogenesis. We found a strong increase in plasma FGF21 levels in response to low dietary protein intake in aged mice, accompanied by an improvement in whole‐body energy homeostasis and WAT thermogenesis. Together, these findings highlight the role of protein restriction in the amelioration of the age‐related decline of adipose function.

## Results

### Effect of dietary protein intake on physical health in young adult and aged mice

**Fig. 1:**
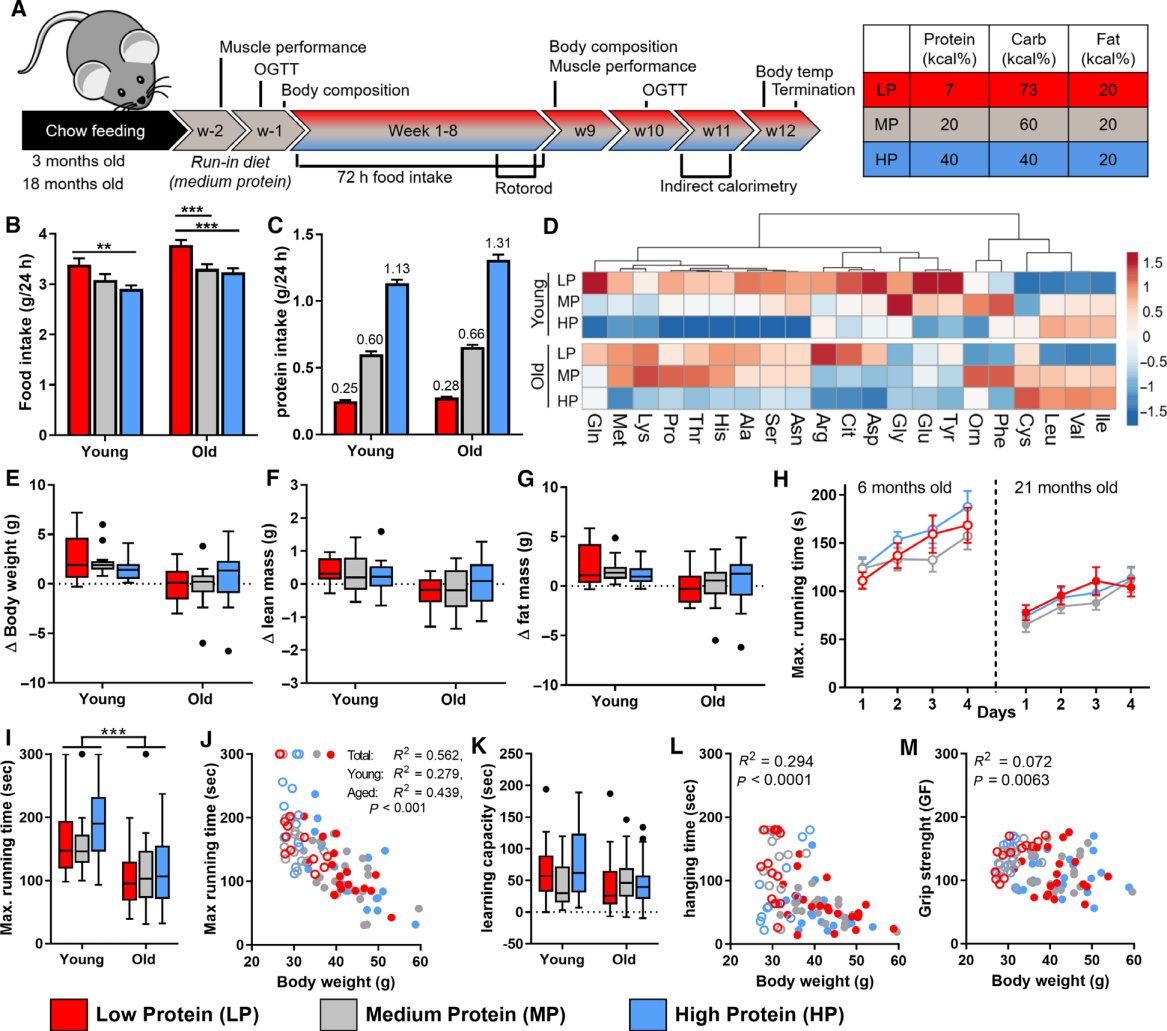
Body weight and other physiological characteristics in young adult and aged mice. (A) Design of the 12‐week dietary intervention and diet composition of the experimental diets. Young adult mice (3 months of age) and aged mice (18 months of age) were normalized on a run‐in diet for 2 weeks to standardize microbial status (young; *n* = 13, old; *n* = 19–22). (B) Food intake, averaged of several measurements in 8 weeks. (C) Protein intake based on individual differences in food intake. (D) Heat map of average amino acid levels in different experimental groups (young; *n* = 8, old; *n* = 12). (E) Changes in body weight, lean mass (F) and fat mass (G) after 8 weeks of the dietary treatment (young; *n* = 13, old; *n* = 19–22). Physical performance measured by rotarod test over four consecutive days (H), with maximal running time over day 2/3/4 (I) and learning capacity (K). Correlation plot between body weight and maximal running time (J) (young; *n* = 13, old; *n* = 19–22). Correlation plots between body weight and hanging time (L) or grip strength (M), measured after 8 weeks of the experimental diet (young; *n* = 13, old; *n* = 19–22). Results are shown as Tukey's box plots or as mean ± SEM (B,C,H). In all graphs, LP = red, MP = grey and HP = blue. Statistical significance was determined by 2‐way ANOVA, followed by Tukey's multiple comparisons tests and indicated as *P < 0.05, **P < 0.01 and ***P < 0.001. In the correlation plot, young adult mice are represented by open symbols, while aged mice are represented by solid symbols. Data were analysed using a nonparametric Spearman correlation.

To evaluate the effects of dietary protein content on metabolic health in the context of ageing, young adult (3 months old) and aged (18 months old) mice were given an isocaloric diet (in which protein was replaced by starch, a complex carbohydrate) with either a reduced protein content (LP, 7%) or an elevated protein content (HP, 40%) compared to a control diet with medium‐protein content (MP, 20%) (Fig. 1A). Over the course of 8 weeks, total intake of the LP diet was higher in both young adult and aged mice (10.0%, *P* = 0.11, and 14.4%, *P* < 0.001, compared with MP) (Fig. 1B). As a result of increased intake, absolute protein intake was effectively reduced by 58.7% in young adult mice and by 57.7% in the aged mice on an LP diet (Fig. 1C). An HP diet did not affect food intake significantly, thereby these mice consumed on average 188% (young adult mice) and 198% (aged mice) of protein, compared with littermates on an MP diet. Despite these substantial changes in protein intake, in LP‐fed animals, plasma levels of all essential amino acids were maintained or even increased, except for the branched‐chain amino acids (BCAA), which were reduced (Fig. 1D).

An important consequence of ageing is the increased risk of sarcopenia, a risk that is elevated due to reduced protein supply. To prevent the decline of muscle mass, protein supplementation is often given to elderly patients [31, 32, 33]. To assess whether reduced protein intake, in our study, negatively affected physical strength in aged mice, we measured three markers of physical health and found no negative consequences. First, either LP or HP did not substantially affect body weight and body composition throughout the experiment. Aged mice did not lose weight on either LP or HP diet, whereas young adult mice displayed normal weight gain (Fig. 1E). In addition, both lean and fat mass were not affected by either an LP or HP diet (Fig. 1F,G, Table 1). Second, muscle performance tests showed an age‐related decline of muscle function, but these changes were not associated with dietary protein content. Physical performance, measured by the maximal running time on an accelerating rotarod, was significantly higher in young adult animals compared with aged animals (Fig. 1H,I), but these were mainly associated with differences in body weight (*R* ^2^ = 0.562, *P* < 0.001), with no significant effect of the diet (Fig. 1J). The motor skill learning capacity was measured over four consecutive days and did not significantly change as a function of age or diet (Fig. 1K). Similar results were obtained with the hanging wire test and the grip strength test, in which age‐related or body weight‐dependent effects were observed, but no effect of dietary protein content (Table 1, Fig. 1L,M). Third, we found no significant effect of dietary protein content on muscle mitochondrial content as determined by citrate synthase activity or mitochondrial function measured by maximal oxidative capacity on different substrates (Table 2). Together, these findings indicate that overall physical health was not significantly affected by a reduced dietary protein intake in either young adult or aged mice and high‐protein (HP) intake in aged mice did not prevent an age‐related decline in physical strength.

**Table 1:** Physiological characteristics in young adult and aged mice. Body weight and composition were determined after 8 weeks of the experimental diets. Physical capacity of young adult and aged mice were measured before and after 8 weeks of the experimental diet using the grip strength test and hanging wire test. Young adult animals: *n* = 13 and aged animals: *n* = 19–22.

	Young animals			Aged animals		
	LP	MP	HP	LP	MP	HP
Body composition						
Body weight (g)	31.2 ± 1.18	30.8 ± 0.77	29.7 ± 0.49	41.2 ± 1.18	41.0 ± 1.19	42.4 ± 1.56
Fat mass (g)	5.5 ± 0.9	4.8 ± 0.5	4.5 ± 0.3	11.4 ± 1.1	11.5 ± 1.0	11.9 ± 1.3
(% of BW)	16.9 ± 2.02	15.2 ± 1.30	15.0 ± 1.03	26.8 ± 1.87	27.2 ± 1.57	26.7 ± 2.04
Lean mass (g)	21.5 ± 0.27	21.7 ± 0.28	21.1 ± 0.36	25.3 ± 0.40	25.3 ± 0.38	25.9 ± 0.42
(% of BW)	69.7 ± 1.85	70.8 ± 1.27	71.0 ± 0.91	62.1 ± 1.54	62.3 ± 1.33	62.2 ± 1.79
Grip strength test						
Before diet (GF)	147 ± 6.8	148 ± 5.4	142 ± 4.6	123 ± 4.4	124 ± 4.3	123 ± 5.0
After diet (GF)	139 ± 7.4	135 ± 4.5	136 ± 6.9	110 ± 7.9	114 ± 6.5	110 ± 6.3
Hanging wire test						
Before diet (s)	43 ± 2.6	41 ± 3.7	39 ± 3.6	15 ± 1.8	16 ± 1.8	18 ± 2.8
After diet (s)	48 ± 16.1	46 ± 7.2	45 ± 8.4	15 ± 2.9	15 ± 1.1	17 ± 2.1

**Table 2:** Mitochondrial capacity of liver and quadriceps in young adult and aged mice. Citrate synthase activity was measured in tissue homogenate (liver or quadriceps) as a marker of mitochondrial content, expressed as µmol/(min∙mg protein). Maximal oxygen consumption stimulated by different oxidizable substrates was expressed in nmol/(min∙mg mitochondrial protein). PM: pyruvate/malate, PMG: pyruvate/malate/glutamate, P‐CoA palmitoyl‐CoA/malate/carnitine. Young adult animals: *n* = 5–6 and aged animals: *n* = 8–10.

	Young animals			Aged animals		
	LP	MP	HP	LP	MP	HP
Quadriceps						
Citrate synthase	0.42 ± 0.03	0.46 ± 0.04	0.47 ± 0.02	0.43 ± 0.02	0.44 ± 0.01	0.46 ± 0.01
PM	132 ± 9.1	168 ± 14.7	163 ± 10.3	146 ± 11.3	165 ± 16.7	166 ± 13.8
PMG	162 ± 9.9	185 ± 17	175 ± 11.5	157 ± 11.4	181 ± 16.8	181 ± 15.5
P‐CoA	50 ± 5.9	49 ± 7.8	60 ± 10.3	50 ± 3	59 ± 5	53 ± 5
Liver						
Citrate synthase	0.12 ± 0.04	0.09 ± 0.01	0.11 ± 0	0.09 ± 0.01	0.13 ± 0.01	0.12 ± 0.01
PM	16.1 ± 2.4^a^	9.2 ± 0.9	9.8 ± 0.7^c^	13.1 ± 1.1	11.6 ± 0.8	9.2 ± 0.8^b^
PMG	49 ± 3.5	39 ± 6.9	44 ± 5	41 ± 3.1	50 ± 4.5	43 ± 6.8
P‐CoA	35 ± 1.8	23 ± 4.5	27 ± 1.4	29 ± 3.2	37 ± 4.3	25 ± 5

^a^ Significant compared with MP, *p* <0.01.

^b^ Significant compared with MP, *p* < 0.05.

c Significant LP vs. HP, *p* <0.01.

### Effects of reduced dietary protein intake on glucose homeostasis

**Fig. 2:**
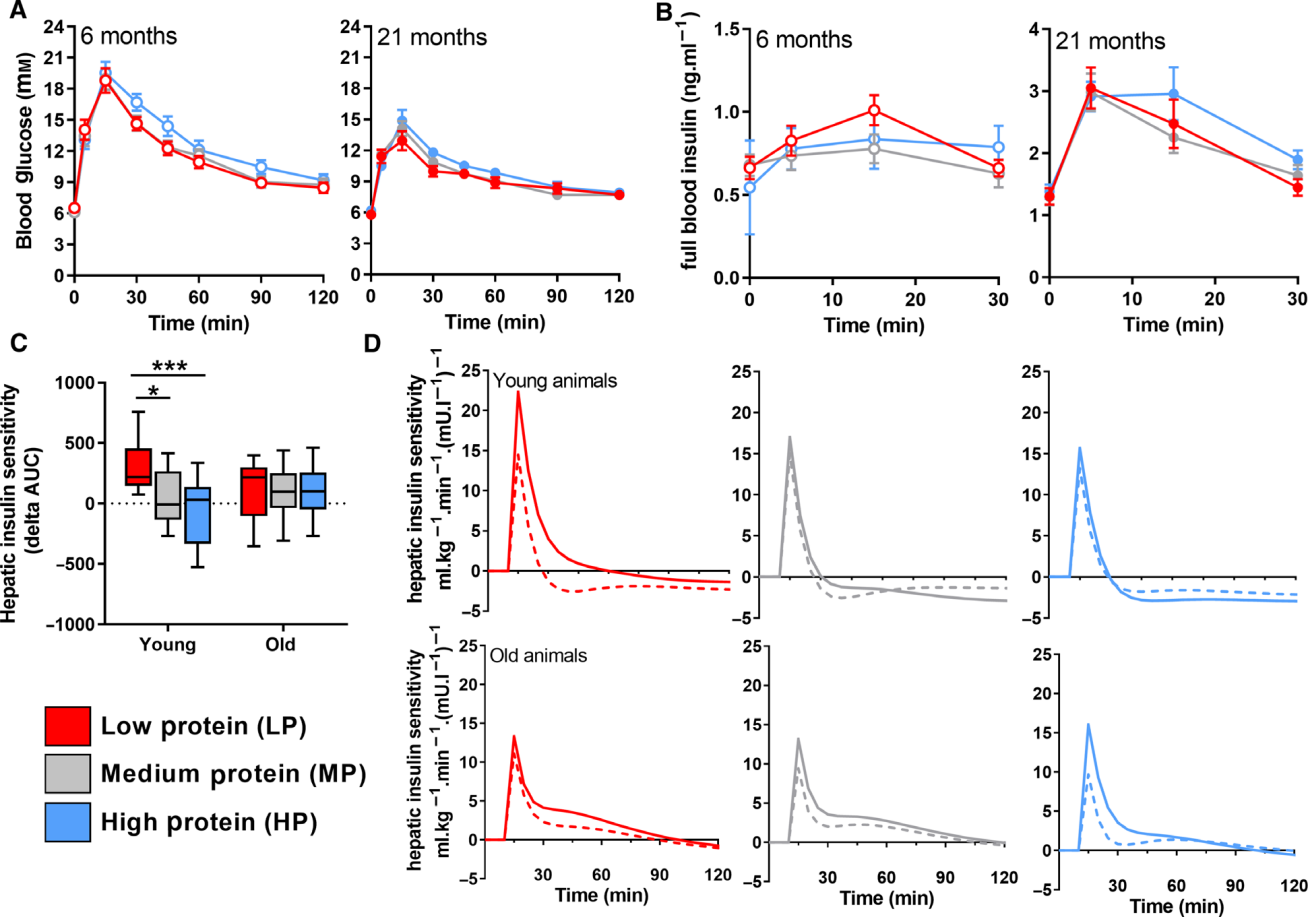
Glucose tolerance and hepatic insulin sensitivity in young adult and aged mice. Glucose tolerance (A) and insulin response (B) after oral administration of 1.5 g·kg^−1^ BW D‐glucose (young; *n* = 13, old; *n* = 19). (C) Hepatic insulin sensitivity, before (dashed line) and after (solid line) 10 weeks of the experimental diet. (D) Hepatic insulin sensitivity, delta AUC (young; *n* = 13, old; *n* = 19). Results are shown as Tukey's box plots (D), as mean (C) or as mean ± SEM (A,B). In all graphs, LP = red, MP = grey and HP = blue. Young adult mice are represented by open symbols, while aged mice are represented by solid symbols. Statistical significance was determined by 2‐way ANOVA, followed by Tukey's multiple comparisons tests and indicated as *P < 0.05, and ***P < 0.001.

As previous studies have reported improved glucose homeostasis as a result of (periodic) short‐term low‐protein feeding [10, 11, 34, 35], we examined the effect of dietary protein intake on glucose homeostasis. Surprisingly, we did not observe major changes in fasting glucose or insulin levels, glucose tolerance or glucose‐stimulated insulin secretion after LP feeding in either young adult or aged mice (Fig. 2A,B, Table 3). Only a small improvement in hepatic insulin sensitivity in young adult mice on an LP diet was observed by tracer‐based glucose kinetics (Fig. 2C,D). In line with this, mitochondrial oxidative capacity of the liver to oxidize pyruvate in the presence of malate was improved in young adult mice on an LP diet (Table 2). Overall, these results show that dietary protein intake does not have a major effect on glucose homeostasis in mice with a stable lean mass and body weight.

**Table 3:** Effects of diet and age on the response to an OGTT. Mice were given oral administration of 1.5 g·kg^−1^ body weight d‐glucose after an overnight (10 h) fast. The AUC was calculated for both glucose and insulin. Young adult animals: *n* = 13 and aged animals: *n* = 19–22.

	3 months	6 month	18 months	21 months
	Before exp	After exp	Before exp	After exp
Fasting glucose				
LP	6.4 ± 0.4	6.7 ± 0.5	6.5 ± 0.4	5.8 ± 0.4
MP	6.0 ± 0.3	6.2 ± 0.5	6.6 ± 0.4	5.9 ± 0.3
HP	6.7 ± 0.5	6.4 ± 0.4	6.2 ± 0.3	6.2 ± 0.3
AUC glucose				
LP	1443 ± 49	1403 ± 65	1278 ± 64	1124 ± 57
MP	1381 ± 59	1418 ± 80	1334 ± 48	1139 ± 36
HP	1472 ± 60	1551 ± 87	1318 ± 66	1210 ± 51
Fasting insulin				
LP	0.7 ± 0.10	0.7 ± 0.07	1 ± 0.14	1.4 ± 0.15
MP	0.6 ± 0.13	0.7 ± 0.07	1 ± 0.15	1.3 ± 0.14
HP	0.8 ± 0.28	0.6 ± 0.28	0.8 ± 0.09	1.3 ± 0.13
AUC insulin				
LP	28.3 ± 3.9	25.4 ± 2.0	50.7 ± 5.2	70.2 ± 6.9
MP	26.9 ± 5.2	23.5 ± 2.2	45.1 ± 5.3	66.0 ± 6.3
HP	22.4 ± 2.3	23.6 ± 4.0	47.6 ± 3.9	67.0 ± 9.8

### Effects of dietary protein intake on energy expenditure and body temperature

**Fig. 3:**
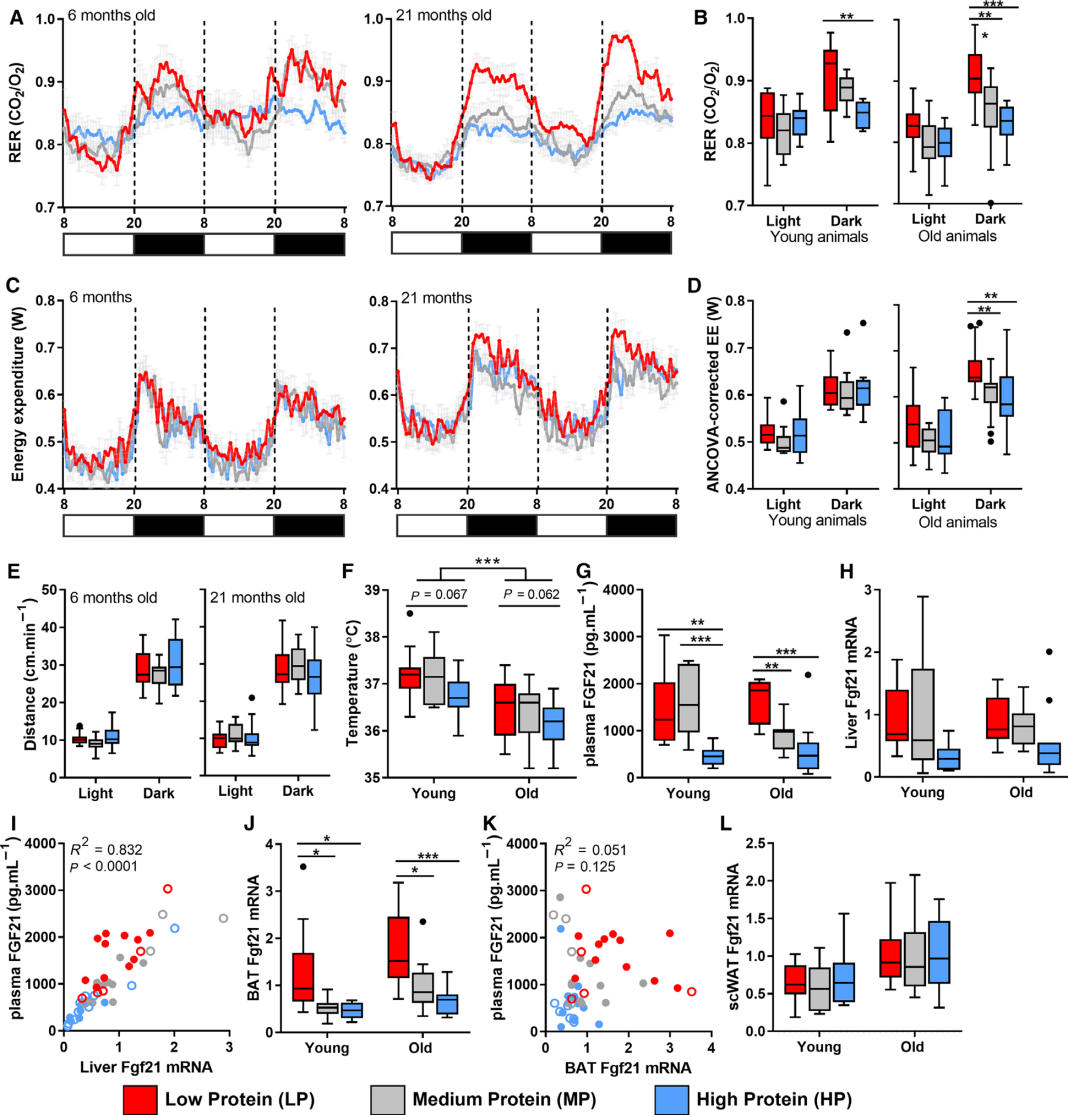
Diet‐induced changes in energy homeostasis and body temperature in aged mice. RER of 48 h in young adult and aged animals (A), average RER of both light and dark phases (B) (young; *n* = 10, old; *n* = 17). (C) EE over 48 h in both young adult and aged mice (young; *n* = 10, old; *n* = 17). (D) ANCOVA‐corrected EE of both light and dark phases in aged mice (adjusted body weight = 37.88 g). (E) Average locomotor activity (cm·min^−1^) during both light and dark phases in young adult and aged mice (young; *n* = 10, old; *n* = 17). (F) Body temperature, measured subcutaneously during the light phase (young; *n* = 10, old; *n* = 17). (G) Plasma FGF21 levels in plasma (young; *n* = 8, old; *n* = 12). *Fgf21* expression levels in liver (H), BAT (J) and scWAT (L) (young; *n* = 9, old; *n* = 12). Correlation plot between plasma FGF21 levels and *Fgf21* mRNA levels in liver (I) and BAT (K) (young; *n* = 7, old; *n* = 10). Results are shown as Tukey's box plots or as mean ± SEM (A,C). In all graphs, LP = red, MP = grey and HP = blue. Statistical significance was determined by 2‐way ANOVA, followed by Tukey's multiple comparisons tests and indicated as *P < 0.05, **P < 0.01 and ***P < 0.001. In the correlation plots, young adult mice are represented by open symbols, while aged mice are represented by solid symbols. Data were analysed using a nonparametric Spearman correlation.

Next, we examined the effect of dietary protein content on whole‐body energy metabolism by indirect calorimetry. The respiratory exchange ratio (RER) during the dark phase was significantly increased due to LP feeding in both young adult and aged mice, indicating a shift in metabolism towards increased glucose utilization (Fig. 3A,B). In contrast to previous studies [13, 22], EE was not significantly increased in young adult mice after LP feeding (Fig. 3C). However, an LP diet substantially affected EE in aged mice. Analysis of the EE data with body weight as covariant (ANCOVA) demonstrated a significant LP‐induced increase in EE in aged mice during the dark phase compared with both MP and HP (Fig. 3D). An HP diet did not affect EE in both young adult and aged mice. Furthermore, an LP diet did not significantly affect voluntary activity, suggesting that the increased EE was due to an enhanced basal metabolic rate (Fig. 3E). In line with this, we found an age‐related reduction in body temperature (*P* < 0.001) and a moderate increase by reduced protein intake (*P* = 0.067 and *P* = 0.062 for LP compared with HP diet in young adult and aged mice, respectively) (Fig. 3F). Together, these results suggest that EE, and to a lesser extent body temperature, can be ameliorated by reduced dietary protein intake in aged mice.

### Effects of low dietary protein intake on expression and circulating levels of Fgf21

Previously, it has been shown that the beneficial effects of protein restriction are mediated by increased hepatic expression and circulating levels of FGF21 [13, 22, 23, 24]. In our study, we also observed an increase in plasma FGF21 levels by an LP diet in the aged mice [average 1638 pg·mL^−1^ (LP) vs. 904 pg·mL^−1^ (MP)], whereas young adult mice already displayed relatively high levels on an MP diet [average 1453 pg·mL^−1^ (LP) vs. 1610 pg·mL^−1^ (MP)] (Fig. 3G). Conversely, an HP diet reduced FGF21 levels in both young adult and aged mice as compared to MP‐fed littermates.

While FGF21 is primarily produced by the liver, in response to metabolic challenges such as fasting and starvation [13], BAT and WAT have also been shown to produce FGF21 in response to cold exposure [36, 37]. In the current study, we observed an effect of reduced protein intake on *Fgf21* in both liver and BAT. Correlation analysis indicates that 83.2% of the variation in plasma FGF21 can be explained by stimulation of hepatic *Fgf21* mRNA expression (Fig. 3H,I). In contrast, this induction of *Fgf21* in BAT did not correlate with plasma FGF21 levels (Fig. 3J,K). No changes in *Fgf21* expression were observed in subcutaneous WAT in response to an LP diet in either young adult or aged mice (Fig. 3L). In conclusion, reduced dietary protein intake increases plasma levels of FGF21 in aged mice and this is primarily caused by an induction of hepatic *Fgf21* expression.

### Effects of dietary protein restriction and age on brown adipose tissue

**Fig. 4:**
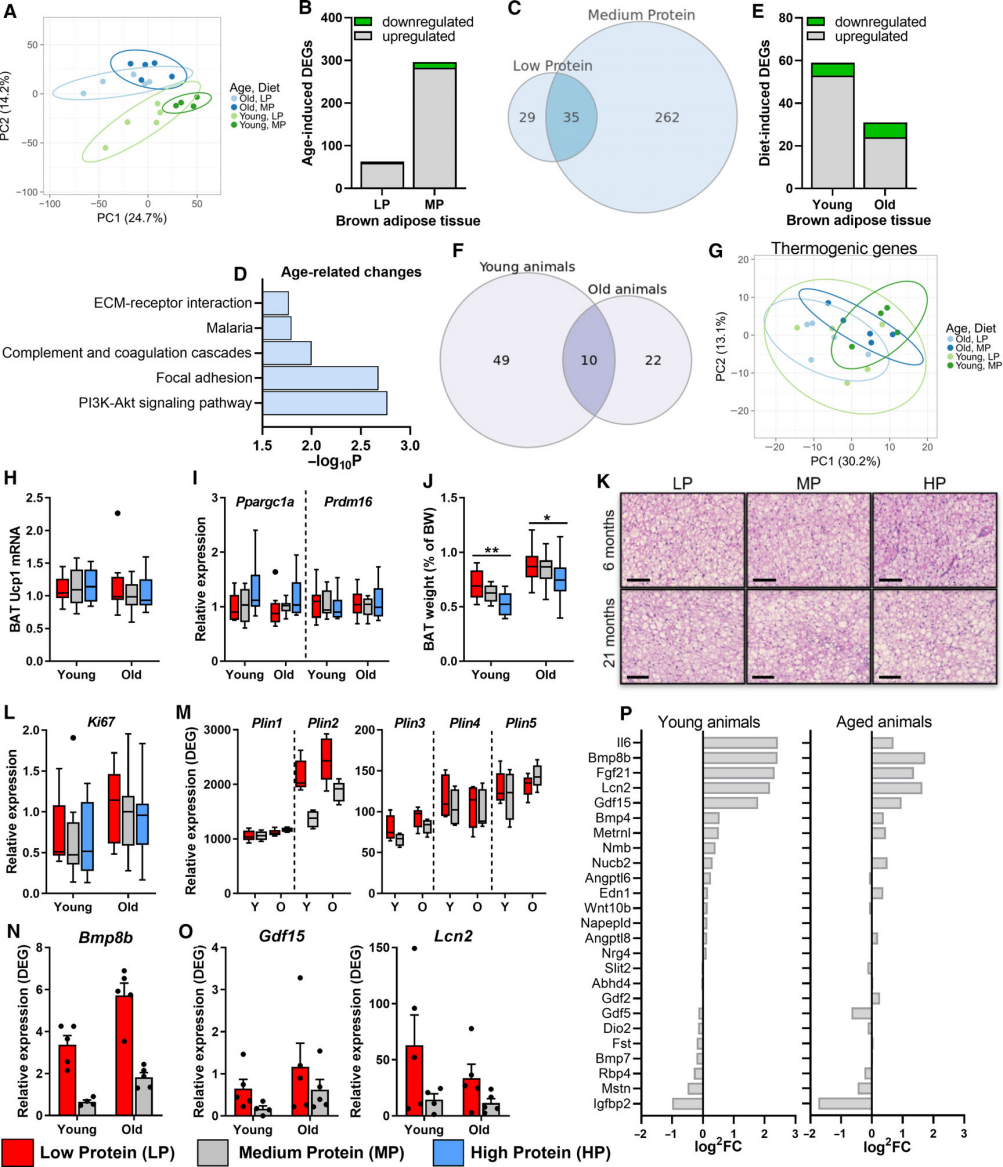
Activation of BAT secretory peptide profile by reduced protein intake. (A) PCA plot, plotting all genes with a significant difference (*P* < 0.05) in either a diet‐ or ageing‐induced comparison (3791 genes) (*n* = 4–5). (B) Number of significant DEGs (*P*_adj_ < 0.1) either upregulated or downregulated in aged mice compared with young adult mice (*n* = 4–5), with a Venn diagram showing overlap between different diets (C). (D) KEGG pathway analysis using DEGs (*P*_adj_ < 0.1) of age‐related changes on an MP diet. (E) Number of significant DEGs (*P*_adj_ < 0.1) either upregulated or downregulated by an LP diet compared with MP diet (*n* = 4–5), with a Venn diagram showing overlap between ages (F). (G) PCA plot, plotting 225 genes involved in KEGG pathway ‘Thermogenesis’ (mmu04714) (*n* = 4–5). mRNA expression levels of *Ucp1* (H) and *Ppargc1a*/*Prdm16* (I) and *Ki67* (L) in BAT (young; *n* = 9, old; *n* = 12). (J) BAT weight, as percentage of total body weight (young; *n* = 13, old; *n* = 19–22). (K) Representative pictures of BAT morphology; scale bar represents 100 µm (*n* = 4). RNA‐seq expression levels of different Plin genes (M), *Bmp8b* (N) *Lcn2* and *Gdf15* (O) (*n* = 4–5). (P) RNAseq batokine expression profile, log^2^ fold change (log_2_FC) differences between LP‐ and MP‐fed animals. Results are shown as Tukey's box plots or mean ± SEM (N, O), with LP = red, MP = grey and HP = blue. Statistical significance was determined by 2‐way ANOVA, followed by Tukey's multiple comparisons tests and indicated as *P < 0.05 and **P < 0.01. In PCA plots, aged LP = light blue, aged MP = dark blue, young adult LP = light green and young adult MP = dark green.

The increased EE, body temperature and BAT *Fgf21* mRNA levels in response to an LP diet pointed towards changes in BAT activity. To investigate the effect of an LP diet on gene expression in BAT, we performed transcriptome analysis. PCA of all differentially expressed genes (DEGs, *P* < 0.05) indicated that the age‐related effect was more profound than the diet‐induced effect on gene expression (Fig. 4A). Interestingly, an LP diet increased the variation in gene expression of both young adult and aged mice (Fig. 3A), resulting in 297 and 64 (35 common) age‐related DEGs [*P* < 0.05, false discovery rate (FDR) = 0.1] for, respectively, MP and LP diets, the majority of which were upregulated (Fig. 4B,C). Genes affected by age were associated with low‐grade inflammation, such as increased levels of the complement system, rather than downregulation of specific genes involved in BAT function (Fig. 4D). Reduced dietary protein intake resulted in 59 and 32 (10 common) DEGs in, respectively, young adult and aged mice (Fig. 4E,F). No effect, however, was observed by an LP diet on the expression of thermogenic genes, including *Ucp1*, peroxisome proliferator‐activated receptor gamma coactivator 1‐alpha (*Ppargc1α*) and PR domain containing 16 (*Prdm16*) (Fig. 4G,I), indicating that thermogenesis was not affected.

An alternative explanation for the observed increase in EE and body temperature could be the increased size of BAT and increased clearance of glucose and nonesterified fatty acids (NEFA) [23]. While reduced dietary protein intake slightly increased BAT weight in both young adult and aged mice (Fig. 4J), we did not detect any stimulation of proliferation, as indicated by the levels of Ki67 mRNA (Fig. 4L). In contrast, we observed minor differences in BAT morphology (Fig. 4K) associated with increased levels of the lipid droplet marker Plin (Fig. 4M), suggesting differences in BAT were most likely due to lipid accumulation. Interestingly, we found several other batokines next to *Fgf21* increased in response to an LP diet, including *Bmp8b* (increased 5.2‐fold and 3.1‐fold in young adult and aged mice, respectively), *Lcn2* and *Gdf15* (> 3‐fold increased expression levels after LP feeding) (Fig. 4N,O). Other genes encoding batokines, however, such as *Angplt8*, *Nrg4*, *Bmp4* and *Bmp7,* were not affected (Fig. 4P). In conclusion, while we found no clear evidence for the increased thermogenic capacity of BAT other than increased BAT weight, an LP diet did increase the expression of selected batokines.

### Effects of dietary protein intake and age on subcutaneous white adipose tissue

**Fig. 5:**
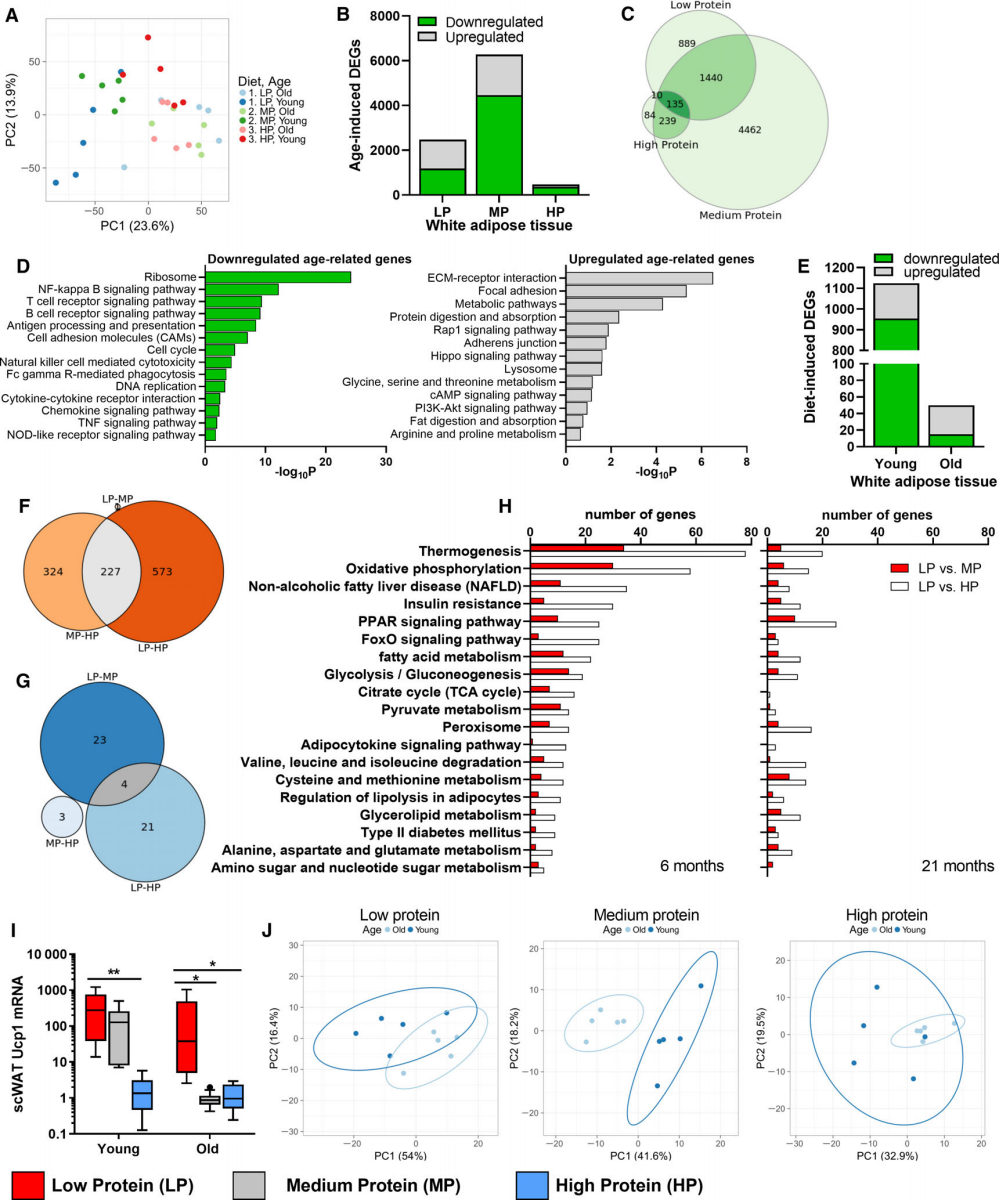
Increased thermogenesis in subcutaneous WAT by reduced protein intake. (A) PCA plot, plotting all genes with a significant difference (*P*_adj_ < 0.1) in either a diet‐ or ageing‐induced comparison (7447 genes), LP = blue, MP = green and HP = red, and aged mice are shown in light while young adult animals are represented in dark colours (*n* = 5). (B) Number of significant DEGs (*P*_adj_ < 0.1) either upregulated or downregulated in aged mice compared with young adult mice, with a Venn diagram showing overlap between different diets (C). (D) KEGG pathway analysis using downregulated DEGs (*P*_adj_ < 0.1; green) or upregulated DEGs (*P*_adj_ < 0.1; red) between young adult and aged animals on an MP diet. (E) Significant DEGs (*P*_adj_ < 0.1) either upregulated or downregulated by any dietary interaction, with a Venn diagram showing overlap between the specific dietary comparisons in young adult mice (F) and aged mice (G). (H) Manual pathway analysis, using all genes significant (*P* < 0.05) in KEGG‐defined pathways, differences between LP‐MP in red and LP‐HP in white. (I) mRNA expression levels of *Ucp1*, LP = red, MP = grey and HP = blue (young; *n* = 8, old; *n* = 12). (J) PCA plots, plotting 225 genes involved in KEGG pathway ‘Thermogenesis’, comparing ages at an LP, MP and HP diets, young adult mice in dark blue and aged mice in light blue (*n* = 5). Statistical significance was determined by 2‐way ANOVA, followed by Tukey's multiple comparisons tests and indicated as *P < 0.05 and **P < 0.01.

In the absence of major effects on BAT, we investigated whether the observed metabolic improvements could be mediated by changes in scWAT. We hypothesized that increased EE could be the result of browning of scWAT, mediated by FGF21. When analysing the transcriptome of scWAT, similar to BAT, the age‐related effects on gene expression were more pronounced than the diet‐induced effects (Fig. 5A). Most age‐related changes in gene expression were observed on an MP diet, while switching diets to either an LP or HP diet resulted in increased individual variation, reducing the number of significant DEGs (Fig. 5B). On an MP diet, most genes found were downregulated by ageing, resulting in highly enriched KEGG pathways related to ribosomal function or innate immune cell signalling. In contrast, upregulated genes were involved in ECM‐receptor interactions, focal adhesion and metabolic pathways (Fig. 5C,D).

Diet changed in total 1124 DEGs significantly in young adult mice, most of which were the result of HP feeding (Fig. 5E,F). In contrast, most of the 50 DEGs significantly changed in aged mice were LP‐mediated (Fig. 5E‐G). Pathway enrichment analysis highlighted changes in the thermogenic pathway (KEGG mmu04714), oxidative phosphorylation, PPARγ signalling and TCA cycle, as a result of LP feeding (Fig. 5H). Further, increased levels of *Ucp1* mRNA levels, an essential gene in adaptive thermogenesis, indicated upregulation of the thermogenic capacity of scWAT. *Ucp1* levels were significantly increased in aged mice by an LP diet compared with both MP and HP diets (Fig. 5I). Noticeably, young adult mice on either an LP or MP diet already exhibited high levels of *Ucp1* compared with mice on an HP diet. In line with these results, transcriptome data show both age‐related and diet‐induced changes in the total thermogenic gene profile (Fig. 5J). A clear age‐induced difference in the thermogenic gene profile of MP‐fed young adult and aged mice was found. However, when fed an LP diet, we found a greater resemblance between young adult and aged mice, in line with a reversal of the age‐related decline in thermogenic capacity. In contrast, an HP diet increased the individual variation between young adult mice, and combining these results with reduced *Ucp1* mRNA expression (Fig. 5I,J), pointed to a low thermogenic potential. Altogether, these results suggest regulation of the thermogenic potential by dietary protein intake.

### Effects of reduced dietary protein intake on age‐related browning of scWAT

**Fig. 6:**
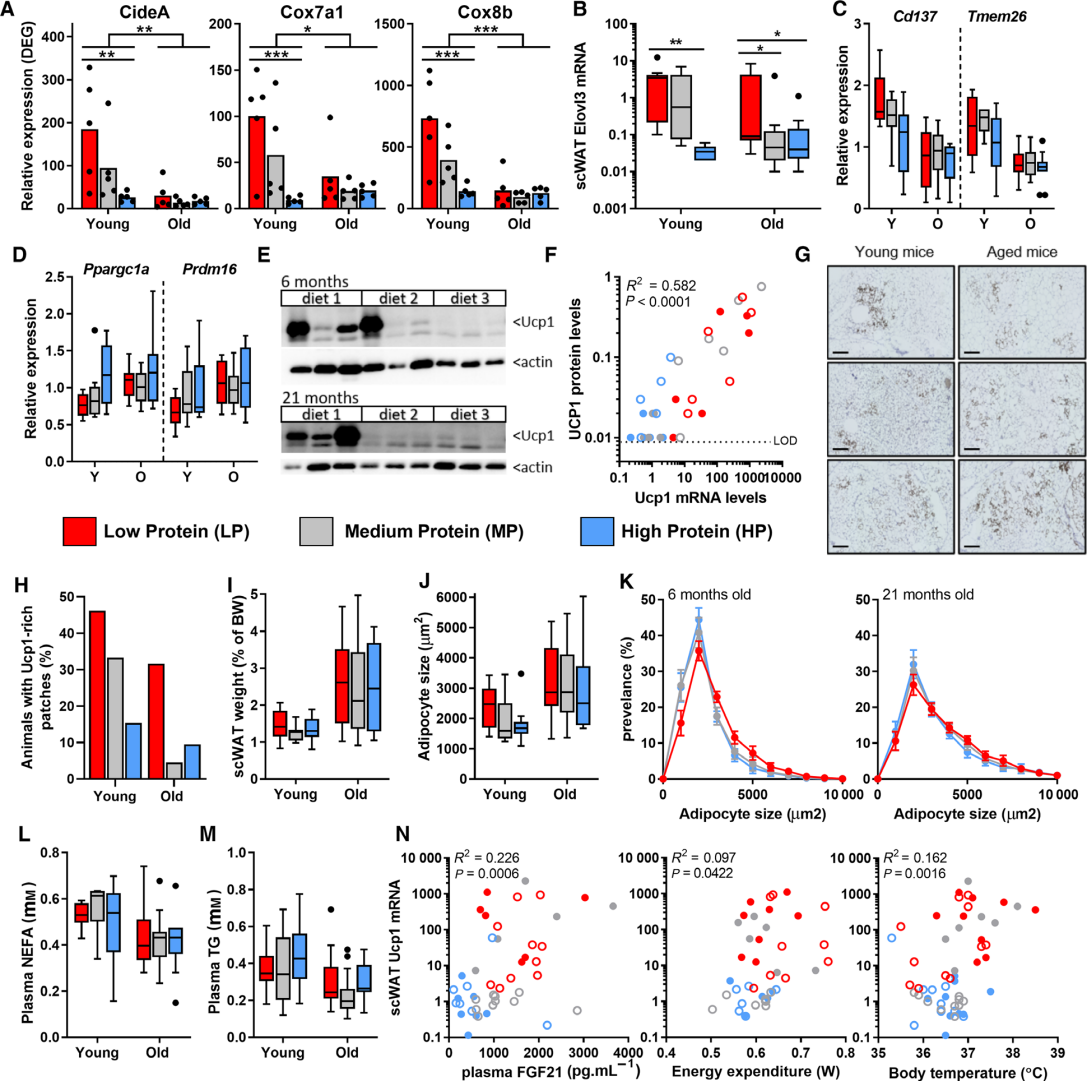
Selective induction of browning of subcutaneous WAT. (A) RNA‐seq expression levels of *Cidea*, *Cox7a1* and *Cox8b* (*n* = 5). mRNA expression levels of *Elovl3* (B) *Cd137*/*Tmem26* (C) and *Pppargc1a*/*Prdm16* (D) (young; *n* = 9, old; *n* = 12). (E) Representative western blots of UCP1 protein expression in scWAT (*n* = 6). (F) Correlation plot between mRNA levels and protein levels of UCP1 (*n* = 5–6). (G) Representative pictures of UCP1 staining showing patches of UCP1‐rich cells in young adult and aged mice on a LP diet; scale bar represents 200 µm, with quantification of the number of animals expressing certain patches (young; *n* = 13, old; *n* = 19–22) (H). (I) scWAT weight, as percentage of total body weight (young; *n* = 13, old; *n* = 19–22). Average adipocyte size (J) and distribution (K) of both young adult and aged mice quantifying size of > 100 adipocytes/mouse (young; *n* = 10, old; *n* = 13). (L) Plasma NEFA of terminal blood sample, fasted for 2–6 h (young; *n* = 8, old; *n* = 15). (M) Plasma TG of terminal blood sample, fasted for 2–6 h (young; *n* = 13, old; *n* = 20). (N) Correlation plots between *Ucp1* mRNA levels and plasma FGF21, EE and body temperature (young; *n* = 6–8, old; *n* = 9–11). Results are shown as mean (H), mean ± SEM (A,K) or Tukey's box plots. In all graphs, LP = red, MP = grey and HP = blue. Statistical significance was determined by 2‐way ANOVA, followed by Tukey's multiple comparisons tests and indicated as *P < 0.05, **P < 0.01 and ***P < 0.001. In the correlation plots, young adult mice are represented by open symbols, while aged mice are represented by solid symbols. Data were analysed using a nonparametric Spearman correlation.

A rapid age‐related decline of the thermogenic capacity of scWAT, as early as 4 months of age, has been previously shown in mice, including a loss of *Ucp1* expression and reduced expression of other thermogenic markers such as *Cidea*, *Cox7a1* and *Cox8b* [29]. Similarly, we found an age‐related downregulation of these genes under MP conditions (Fig. 6A). We further investigated the browning capacity of the scWAT and found a strongly increased expression of the beiging‐associated gene *Elovl3* due to LP feeding, to the same extent as *Ucp1* (Fig. 6B). However, other beiging‐related genes, such as *Cd137* (*Tnfrsf9*) and *Tmem26,* only showed a trend towards an increase in young adult LP‐fed mice compared with HP‐fed mice (Fig. 6C). In contrast, classical brown adipocyte‐associated genes such as *Prdm16* and *Ppargc1a* were not altered in scWAT (Fig. 6D).

To assess whether changes in *Ucp1* mRNA levels were also accompanied by higher UCP1 protein levels, we determined protein levels by western analysis and found a positive correlation with *Ucp1* mRNA levels (*R* ^2^ = 58,2%, *P* < 0.0001, Fig. 6E,F). Closer examination of the adipose tissue by UCP1 staining revealed heterogeneity of scWAT, with sporadic patches of brown‐like adipocytes across the WAT. In animals on an LP diet, different locations in the tissue contained a high number of cells with multiple lipid droplets and high expression of UCP1 protein (representative picture in Fig. 6G). The UCP1‐rich patches were more frequently present in young adult animals compared with the aged animals (Fig. 6H). However, an LP diet was able to increase the number of these patches in aged mice to levels similar to that of young adult mice.

In contrast to these patches of UCP1‐rich cells, the surrounding nonthermogenic adipocytes did not change in morphology. Increased thermogenesis could potentially affect overall lipid oxidation, adipocyte size and production of healthy adipokines. However, we found no differences in scWAT mass, adipocyte size or distribution upon analysis of representative pictures of nonthermogenic zones (Fig. 6I–K). In addition, pathway analysis revealed that other functions such as adipokine signalling and lipolysis were not significantly altered in the total scWAT depot (Fig. 5H). In line with this, plasma NEFAs and TG were also not significantly affected by protein restriction (Fig. 6L,M).

Taken together, while the thermogenic capacity of scWAT shows an age‐related decline, this effect could be largely reversed by an LP diet. Spearman's correlations connect *Ucp1* mRNA levels to plasma FGF21 levels, EE and body temperature, suggesting that the reversal of the age‐related decline in metabolic health starts with activating the thermogenic capacity in scWAT (plasma FGF21 levels; *R* ^2^ = 22.6%, *P* = 0.0006, EE; *R* ^2^ = 9.7%, *P* = 0.042, body temperature; *R* ^2^ = 16.2%, *P* = 0.002) (Fig. 6N). Overall, these data suggest a localized browning in the scWAT, affecting age‐related changes in EE and body temperature.

## Discussion

In Western societies, the percentage of people over the age of 65 is rapidly increasing. With age, also the risk of obesity increases, and approximately one‐third of the population develops obesity by the age of 60–70 [38]. Ageing and obesity are prominent risk factors for many metabolic diseases; therefore, understanding how ageing negatively affects energy metabolism and identifying strategies to reduce age‐related decline in metabolic health are imperative. Dietary protein restriction has been shown to improve metabolic health in multiple species, including mice and humans [5, 7, 10, 13]. Long‐term dietary protein restriction in mice, starting directly after weaning, also indicated that longevity can be increased by reduced protein intake [7, 8, 9]. However, whether a short‐term intervention with a low‐protein diet starting at an advanced age also has beneficial effects on metabolic health is not known. Here, we show that short‐term dietary protein restriction in aged mice, starting at the age of 18 months, ameliorated the age‐related decline of some aspects of metabolic health, including EE, circulating levels of FGF21 and browning of scWAT without noticeable adverse effects on physical health. Our findings indicate that interventions based on protein restriction have the potential to improve metabolic health when started at an older age. Previous studies into the impact of a late‐life switch to CR reported either no effect or an increased survival [39, 40, 41]. A recent study using dietary restriction at late‐life in female mice showed no metabolic changes in WAT or BAT, indicating that the body has a memory of earlier nutrition [39]. While these differences in outcome could have been influenced by differences in experimental design, such as starting age, duration of the diet or gender, it also suggests that diet composition is an important factor for the effectiveness of late‐life dietary intervention strategies.

Previously, FGF21 has been implicated in increasing EE and the browning of WAT via brain‐mediated βKlotho signalling [26, 27]. Similarly, we found that plasma FGF21 levels correlated with markers of browning of scWAT in our study. Both *Ucp1* and *Elovl3* mRNA levels in the scWAT were strongly increased by an LP diet. ELOVL3 has been implicated as a key thermogenic enzyme as *Elovl3*‐deficient mice are unable to activate their BAT under conditions of cold exposure due to a decreased capacity to elongate saturated fatty acyl‐CoAs for heat generation [42]. Other beige‐related genes, such as *Cd137* and *Tmem26*, have been implicated as markers of increased thermogenesis in WAT, noted by high expression levels in beige adipocytes compared with brown and white adipocytes and on specific white preadipocytes with great potential activate adipogenesis with high thermogenic capacity [43]. However, in this study, we only observed minor changes in these genes. In addition, classical brown adipocyte‐associated genes, such as *Prdm16* and *Ppargc1a,* are thought to be involved in the recruitment of brown preadipocytes, thereby activating thermogenesis from *Myf5+* precursor cells [44]. In our study, these genes were not altered in the total scWAT depot. As we determined mRNA levels in the total adipose depot, it could still be these genes are upregulated specifically in the Ucp1‐rich patches. Furthermore, the finding of the specific UCP1‐rich patches raises the question whether browning of these locations is due to differentiation of white adipocytes or infiltration of brown adipocytes. Closer examination of the areas in WAT with high *Ucp1* expression is needed to provide further information on the mechanisms of browning.

In addition to increased thermogenesis of scWAT, specific activation of BAT by FGF21 has been shown previously to increase glucose/NEFA uptake, improve insulin sensitivity and increase *Ucp1* expression [22, 23, 26]. However, our LP intervention did not affect overall insulin homeostasis or *Ucp1* expression levels in BAT. Even though we cannot exclude the possibility of increased glucose/NEFA clearance by BAT, our results do not indicate that heat production in BAT is activated. Besides *Fgf21* mRNA expression, we did found increased expression of other adipokines such as *Bmp8b, Lcn2* and *Gdf15* in our transcriptomic data, which can promote the thermogenic potential of BAT and WAT. Whether BAT‐derived FGF21 reaches the circulation remains controversial [36, 45, 46]; however, it could have indirect effects in this study by stimulating batokine production. BMP8b is part of a large family of BMPs that are involved in the differentiation of white and brown adipocytes, with BMP8b contributing to maximal thermogenic capacity by remodelling of the neurovascular network [47, 48]. Similarly, LCN2 and GDF15 are involved in inducing thermogenesis, as suggested by knockout studies and cold‐exposure experiments [49, 50, 51]. However, as the thermogenic potential measured by *Ucp1* expression was not induced in BAT after LP intervention, the role of these batokines in the current study needs to be further evaluated.

Insulin resistance plays a central role in the development of metabolic diseases. Ageing is associated with insulin resistance due to age‐related changes in adipose tissue function, mass and distribution [52, 53]. These changes are accompanied by diminished lipid handling, low‐grade inflammation, defective thermogenesis and impaired *de novo* adipogenesis [28, 29, 30]. In our study, aged mice displayed insulin resistance, increased adiposity and decreased thermogenesis. Short‐term LP feeding did not impact glucose tolerance, insulin sensitivity or adiposity at an advanced age, and only mildly improved hepatic insulin sensitivity in young adult mice, without differences in body weight. In contrast to our observations, some studies have reported improved glucose homeostasis as a result of LP feeding in both (young) mice and humans [10, 11, 34, 35], but these latter findings were accompanied by decreased body weight, potentially explaining the improved insulin sensitivity. Our results, however, show that EE and thermogenesis can be improved by LP feeding in aged mice, indicating that these LP‐induced effects are independent of insulin sensitivity and body weight changes. Interestingly, while aged mice respond well to a 7% low‐protein diet regarding EE, suggesting that EE and insulin sensitivity could be distinct features of protein restriction in aged mice, the effects of LP on EE could not be reproduced in young adult animals. In young mice, food intake is increased as a result of LP feeding, and animals do not gain weight, but changes in EE are not detectable. Subtle changes in EE, nutrient absorption, activity or a combination of these might explain this discrepancy in energy balance; however, a larger cohort is needed to provide answers. In addition, a previous study shows a divergent effect of 5% or 10% protein diets [54], suggesting a threshold of protein intake to improve metabolic parameters. This hypothesis could also explain why young adult mice on a 7% protein diet did not display increased EE. The fact that aged mice did respond to a 7% protein diet suggests that this threshold could be age‐specific.

Epidemiological studies suggest that diets high in protein and low in carbohydrates are associated with increased mortality [55]. Data from the Third National Health and Nutrition Examination Survey (NHANES III), stratified for age indicated that HP diets (> 20%), were associated with increased mortality and cancer in respondents under the age of 65 years, and with a fivefold increase in diabetes mortality across all ages [56]. In respondents over 65, however, HP intake was associated with reduced overall mortality. A possible explanation is that at an advanced age, low‐protein intake might result in a metabolic decline due to decreased availability of amino acids, protein anabolic resistance and immobility. In agreement with the effect of ageing turning the beneficial effects of protein restriction on mortality into negative effects, feeding 2‐year‐old mice a 4% protein diet led to a rapid weight loss of 10% in 15 days [56]. Although not measured, this was likely due to loss of muscle mass, an age‐related risk that is elevated with reduced protein supply [57]. To avoid muscle mass deterioration at an advanced age, our study was conducted with a less restricted protein content of 7%. Dietary protein restriction reduced plasma levels of BCAAs, which are often associated with metabolic diseases [58, 59], but increased levels of most other amino acids, suggesting no signs of amino acid deficiencies. In our study, aged mice displayed no reduction in body weight, lean mass or fat mass during the 3‐month intervention with different protein diets. Physical tests determining muscle mass and function revealed only an age‐related decline and no negative effects of the LP diet, indicating that moderate protein restriction is well‐tolerated at an advanced age in mice. Interestingly, feeding an HP diet for 3 months at advanced age did not ameliorate the age‐related decline in muscle strength, suggesting a discrepancy with the common consensus in literature [31, 32, 33]. However, the short duration of the diet and the age at which the diet is started could have an effect on the outcome. Starting the diet at 18 months of age, when the negative effects on grip strength and hanging wire are still relatively small, the impact of a HP diet may not have been noticeable, suggesting the consequences of protein intake might be age‐specific.

Important to mention is the possible role of carbohydrate intake on the diet‐induced effects on energy metabolism in this study. Firstly, a change in the protein–carbohydrate ratio changes glucose oxidation, resulting in increased RER. However, whether these changes in RER directly affect EE remains unknown. Secondly, carbohydrate intake could also have influenced hepatic *Fgf21* expression by regulation of the transcription factor ChREBP in conditions of high sucrose intake [60, 61]. In our study, the higher sucrose content of the AIN‐93G semi‐synthetic diet compared with chow might indeed have stimulated hepatic *Fgf21* transcription at the start of the experiment. Still, it does not explain the differences between our experimental groups. In our diet, the protein content has been substituted with starch, not sucrose. Therefore, mechanisms underlying *Fgf21* stimulation are unlikely the result of changes in ChREBP regulation by carbohydrate intake. More likely, the amino acid composition affects FGF21 levels and improves metabolic health [10, 34]. Several diets with reduced levels of essential amino acids such as methionine or BCAAs (leucine, isoleucine or valine) mimic the effects of an LP diet [10, 62, 63]. Whereas the BCAAs were significantly reduced in plasma, most other amino acid levels were increased by dietary protein restriction in both ages, which is in line with previous studies [8]. It needs to be further determined whether mechanisms of hepatic *Fgf21* transcription are the result of selective amino acid deprivation.

## Conclusion

Taken together, it is evident that an LP diet provides several benefits on metabolic health in aged mice. Our study indicates that an short‐term intervention with an optimal balance of dietary protein and carbohydrates can positively impact metabolic health in aged individuals as we find increased EE and thermogenesis, via browning of WAT, without any negative reduction in muscle function. We speculate that FGF21‐mediated changes in scWAT are crucial to achieve metabolic health in aged individuals, suggesting that age‐related decline of adipose function can be effectively improved by targeting FGF21 signalling. Altogether, this study highlights the possibility of browning of scWAT to improve metabolic health by LP feeding in the context of advanced age.

## Materials and methods

### Animals

Male C57BL/6J mice (The Jackson Laboratory, Bar Harbor, ME, USA), bred and aged in our facility, were used at 3 months (*n* = 13) and 18 months (*n* = 22) of age in this study. Animals were housed in a light‐ and temperature‐controlled facility (12‐h light cycle, 21 °C room temperature) with free access to water and standard chow (RM1; SDS Diets, Woerden, The Netherlands). Animal experiments were performed with the approval of the National Ethics Committee for Animal Experiments of The Netherlands, in accordance with relevant guidelines and regulations (including laboratory and biosafety regulations). Mice were excluded from the experiment if they reached humane endpoint (15% body weight loss or sustained inactivity) or had developed severe (liver) tumours at the end of the experiment. In total, < 15% of the aged animals were excluded from the study, with no substantial differences between experimental groups.

### Animal experiments

To study the short‐term metabolic effects of protein intake, animals were given a semi‐synthetic diet, varying in protein content, for 12 weeks. Prior to the experiment, mice were given a run‐in diet (20% protein control diet) for 2 weeks, to normalize microbial status. All experimental diets were based on the AIN‐93G breeding diet supplemented with cysteine (D10012G; Open Source Diets, New Brunswick, NJ, USA) containing a fixed fat content of 20% (% of total kcal). Experimental diets consisted of a low‐protein diet (LP; 7% of total kcal), MP diet (control; 20% of total kcal) and HP diet (40% of total kcal), in which protein is replaced by an isocaloric amount of starch. For a detailed description of the diets, see Table S1.

Aged animals (18 months of age) were divided into the experimental groups, normalized for body weight and 4‐h fasting glucose, insulin and cholesterol levels (Fig. S1). Young adult animals (3 months of age) were randomly assigned to one of the three experimental diets. Animals were weighed weekly and body composition was determined by nuclear magnetic resonance (NMR), both during the run‐in period and after 8 weeks of the experimental diet, using the Bruker Minispec LF110 bicinchoninic acid Analyzer (Bruker BioSpin, Rheinstetten, Germany). Food intake was measured several times for a 72‐h period. Two days before termination, mice were injected subcutaneously with a temperature sensor (IPTT300; BMDS, Seaford DE, USA), and body temperature was measured the next day during the light phase. Animals were terminated after a 2‐ to 6‐h fast, introduced 1 h before the lights were turned on. A detailed schematic overview of the experimental design is shown in Fig. 1A.

### Muscle strength, endurance and physical performance tests

The hanging wire test and grip strength test were performed during both the run‐in period and after 8 weeks of the experimental diet to determine changes in physical strength. Physical health and motor skill learning ability were tested using the rotarod test after 7 weeks of the experimental diet.

#### Grip strength test

Forelimb grip strength was measured using Ametek digital force gauge (Chatillon DFE II, Elancourt, France). Mice were positioned to grasp the bar with forelimbs only and then pulled horizontally until letting go. The test was performed six times (two rounds of three trials each). Within rounds, mice were given minimal rest (30 s), while between rounds, mice were returned to their home cage for > 15 min. The maximum grip strength of the six trials was recorded.

#### Hanging wire test

Strength and endurance were tested using the hanging wire test. A wire (3 mm in diameter, 55 cm in length) was suspended between two supports, 35 cm above the tabletop. Mice were positioned at the centre of the wire, hanging by their forelimbs. The test was performed six times (two rounds of three trials each), in which the maximal hanging times were recorded. Within rounds, mice were given minimal rest (30 s), while between rounds, mice were returned to their home cage to rest for > 15 min. Whenever the mouse reached the end of the wire, it was immediately placed back at the original position at the centre and recording time continued. When the mouse turned and fell by its hind limbs, the fall was considered voluntary and was not counted.

#### Rotarod test

Neuromuscular performance was determined with the rotarod apparatus (Series 8; IITC Life Science, Woodland Hills, CA, USA), using a 4‐day protocol. On day 1, mice were trained to run on a rotating rod, with three runs accelerating in speed slowly (1–5 r.p.m. in 180 s, 1–5 r.p.m. in 30 s and 4–10 r.p.m. in 60 s). A fourth run consisted of the experimental protocol with acceleration from 4 to 20 r.p.m. in 180 s. The running time was measured until the mice fell or until 300 s was reached. On days 2, 3 and 4, the experimental procedure was repeated three times, with 15‐min rest between runs. For each day, the maximal running time was recorded. Learning capacity was calculated as the difference between the maximal running time on day 3/4 and the experimental run on training day 1.

### Indirect calorimetry

Real‐time metabolic analyses were performed using a Comprehensive Laboratory Animal Monitoring System (TSE Systems GmbH, Bad Homburg, Germany) at week 11 of the experimental diet. After a period of overnight acclimatization, carbon dioxide (vCO_2_) production, oxygen (vO_2_) consumption, RER, EE, food intake and activity were determined for 48 h in individual mice. Infrared beams recorded locomotor activity according to the number of beam break events in the horizontal (x) and vertical (z) planes.

### Glucose homeostasis

Glucose homeostasis and insulin sensitivity were determined at week 10 of the experimental diet, by performing an oral glucose tolerance test (OGTT) in combination with analysis of tracer‐based glucose kinetics. For this, 1.5 g·kg^−1^ body weight d‐glucose [25% w/v of which 5% w/w stable isotope labelled [6,6‐^2^H_2_]‐glucose tracer (Cambridge Isotope Laboratories, Andover, MA, USA)] was administered orally after an overnight (10 h) fast. At 0, 5, 15, 30, 45, 60, 90 and 120 min after glucose administration, blood glucose levels were determined using an OneTouch Select Plus glucose meter (LifeScan, Zug, Switzerland) and blood spots were collected on filter paper (Sartorius Stedim, TFN 180 g·m^−2^, Nieuwegein, The Netherlands) for tracer analysis [64]. To determine insulin levels, blood spots were collected at 0, 5, 15, 30, 60 and 120 min after glucose administration. Insulin was extracted from the blood spots and determined using the rat insulin ELISA Kit from Crystal Chem and mouse insulin standard (Cat. 90010 and 90020; Zaandam, The Netherlands) according to the manufacturers' protocol. The values for insulin, as measured in bloodspots, were adjusted by factor 1.28, based on matching samples from plasma. Total area under the curve (AUC) was calculated for both glucose and insulin curves, over the periods 0–120 min and 0–30 min, respectively.

Fractional distribution of [6,6‐^2^H_2_]‐glucose was determined by gas chromatography quadrupole mass spectrometry (Agilent 9575C Inert MSD; Agilent Technologies, Amstelveen, The Netherlands) according to van Dijk *et al*. [65]. In short, glucose was extracted from the bloodspot and converted to its pentaacetate derivative. Positive chemical ionization with methane enabled monitoring of ions *m/z* 408–412 (corresponding to m_0_–m_4_ mass isotopologues) which were corrected for the fractional distribution due to natural abundance of ^13^C by multiple linear regression [66] to obtain the excess fractional distribution (M_0_–M_4_) due to the dilution of administrated [6,6‐^2^H_2_]‐glucose; M_2_ represents the fractional contribution of the administered tracer and was used in the calculations of blood glucose kinetics. To describe changes in blood glucose kinetics in mice, we used the minimal model that was adjusted for mice (MiniM) as developed by van Dijk (Data S1). In short, to generate a sufficient number of samples that cannot be taken from mice, we used a mathematical approach using the measured values at indicated time points. The model used allows estimations of peripheral glucose utilization rates, peripheral insulin sensitivity and hepatic insulin sensitivities. Hepatic insulin sensitivity was calculated as a ratio of the endogenous glucose production and the insulin levels over the course of the experiment, and displayed as the delta AUC (*t* = 0–120 min). Relevant equations are shown in the Data S1.

### Muscle mitochondrial content and capacity

To determine mitochondrial content and capacity, mitochondria were isolated from the quadriceps muscle by differential centrifugation as described previously [67]. The O_2_ fluxes in isolated mitochondria were measured using MiR05 buffer (respiration buffer) containing 110 mm sucrose, 60 mm potassium lactobionate, 20 mm taurine, 20 mm HEPES, 0.5 mm EGTA, 10 mm KH_2_PO_4_, 3 mm MgCl_2_, 1 mg·mL^−1^ BSA, pH 7.1 [68] under three conditions, with different oxidizable substrates; (a) 2 mm pyruvate plus 2 mm malate; or (b) 2 mm pyruvate plus 2 mm malate plus 5 mm glutamate; or (c) 25 μm palmitoyl‐CoA plus 2 mm l‐carnitine plus 2 mm malate, at 37 °C using a two‐channel high‐resolution Oroboros Oxygraph‐2k (Oroboros, Innsbruck, Austria). The O_2_ fluxes were normalized for protein content, determined with the bicinchoninic acid Protein Assay Kit (Pierce, Thermo Fisher Scientific Inc., Rockford, IL, USA) and expressed as nmol/(min∙mg mitochondrial protein). Results were corrected for the ratio of citrate synthase activity (described below) in tissue homogenate to the isolated mitochondria, as a reflection of the enrichment of mitochondrial preparations.

Citrate synthase activity was determined, as a marker of mitochondrial content, in isolated mitochondria and quadriceps homogenates (15%, w/v in PBS) in PBS (pH 7.4), by measuring the formation of 5‐thio‐2‐nitrobenzoic acid at 412 nm at 37 °C [69]. In short, the assay mixture contained 0.1 m Tris (pH 8.1), 5 mm triethanolamine/HCl, 0.05 mm EDTA, 0.1% Triton X‐100, 0.5 mm oxaloacetate and 0.1 mm dithionitrobenzoic acid, and the reaction was started with 0.5 mm acetyl‐CoA. Enzyme activities were expressed as μmol/(min∙mg protein).

### Plasma levels of FGF21, free fatty acids, cholesterol and amino acids

Plasma FGF21 levels were determined using the Mouse/Rat Quantikine ELISA (MF2100; R&D Systems, Minneapolis, MN, USA) according to the manufacturers' protocol. Plasma‐free fatty acid levels were determined using the Diasys NEFA FS Kit (#15781; Holzheim, Germany) according to manufacturers’ protocol, measuring absorbance at 540 nm. Plasma cholesterol levels were determined according to manufacturers’ protocol (Roche, 11489232, Mannheim, Germany), using Diasys Cholesterol Standard (#113003010030). Amino acid levels were determined using cation‐exchange high‐performance liquid chromatography followed by postcolumn ninhydrin derivatization, on a Biochrom 30 analyzer (Pharmacia Biotech, Cambridge, UK) [70]. Norleucine was used as an internal standard. A physiological amino acid calibration standard was used for calibration (Sigma‐Aldrich, Darmstadt, Germany).

### Adipose tissue histology and immunohistochemistry

For adipose tissue histology, BAT and subcutaneous white adipose tissue (scWAT) were fixed in 10% formalin overnight and paraffin‐embedded sections (4 μm) were stained with haematoxylin and eosin (H&E). Images of scWAT were taken with Aperio Image Scope (Leica Biosystems, Amsterdam, The Netherlands) for adipocyte size assessment, in which > 100 adipocytes were randomly counted for each mouse, using imagej (NIH, Bethesda, MA, USA).

To assess the thermogenic capacity of adipocytes, H&E staining of four consecutive slides, interspaced with 300–400 μm, was scored for the presence of multilocular cells. To confirm a brown‐like phenotype, UCP1 was detected in paraffin‐embedded scWAT tissues by immunohistochemistry. The endogenous peroxidase activity was blocked with 1% v/v H_2_O_2_ in methanol. After a 15‐min treatment with normal goat serum, sections were incubated overnight at 4 °C with the primary antibody rabbit anti UCP1 (ab10983; Abcam, Cambridge, UK, 1 : 1000 diluted in PBS). For 30 min, the sections were incubated with the secondary antibody goat anti‐rabbit/biotin (BA‐1000; Vector, 1 : 250) and subsequently with VECTASTAIN ABC Reagents (PK4000; Vector Labs, Burlingame, CA, USA) for another 30 min. The reaction was visualized by incubating with 3,3′‐diaminobenzidine solution for 10 min.

### Western analysis

Homogenates of scWAT were prepared using RIPA lysis buffer (50 mm Tris/HCl, 150 mm NaCl, 5 mm EDTA, 30 mm pyrophosphate, 50 mm NaF, 1% (v/v) Triton X‐100, 1 mm PMSF, 2 mm orthovanadate) with Halt protease and phosphatase inhibitor cocktail (Thermo Scientific). Protein levels were determined, normalized using the bicinchoninic acid assay (Pierce) and subjected to a 4–15% gradient SDS/PAGE using the Mini Protean 3 System (Bio‐Rad, Hercules, CA, USA), followed by transfer to poly(vinylidene difluoride) membranes. Standard western analysis was performed using 5% milk in PBS as blocking solution and antibodies to UCP1 [ab10983, 1.3 µg·mL^−1^ (1 : 1000), Abcam] and actin [a2066, 6 µg·mL^−1^ (1 : 100), Sigma, Zwijndrecht, The Netherlands]. HRP‐labelled secondary antibodies were used at 1 : 5000 dilution, and visualization was done by ChemiDoc MP Imaging Systems (Bio‐Rad).

### Gene expression analysis

Total RNA was isolated from mouse liver and adipose tissues using phenol/chloroform extraction with TRIzol (Invitrogen). Transcript levels of synthesized cDNA (M‐MLV reverse transcriptase; Thermo Fisher Scientific, Waltham, MA, USA) were measured with Fast Advance TaqMan Master Mix (Applied Biosystems, Foster City, CA, USA) or Fast Start SYBR Green (Roche) on a QuantStudio 7 Flex Real‐time PCR System (Applied Biosystems). C*_t_* values were normalized by a standard curve and presented as relative expression of housekeeping gene *36B4*. qPCR primers are listed in Tables S2,S3.

### Transcriptome analysis

Total RNA from scWAT and BAT was isolated for RNA sequencing using the RNeasy MINI Kit (Qiagen, Venlo, The Netherlands), according to manufacturer’s protocol. Quality control was performed using a fragment analyser (ProSize 3.0; Advanced Analytical Technologies, Inc., Amstelveen, The Netherlands) confirming all scWAT and BAT samples had an RNA quality number (RQN) value greater than 6 and 8, respectively. A total of 1 µg RNA was used for library preparation and sequencing, performed at Novogene Co. Ltd. Hong Kong using the Illumina HiSeq 2500 instrument on the PE150 platform. Paired‐end clean reads were mapped to the reference genome (mmu_GRCm38.p6) using HISAT2 software. HTSeq was used to count the read numbers mapped of each gene, including known and novel genes. Fragments per kilobase of exon model per million mapped reads of each gene was calculated based on the length of the gene and reads count mapped to this gene, as a means of normalizing for the effect of sequencing depth and gene length.

Differential expression of gene analysis between two groups (*n* = 4–5) was performed using DESeq2 R package, providing statistical routines using a model based on the negative binomial distribution. The resulting *P*‐values were adjusted using Benjamini and Hochberg’s approach for controlling the FDR. Genes with a *P*_adj_ < 0.1 found by DESeq2 were assigned as differentially expressed. For validation of the RNA sequencing data, the expression of several genes was also determined by qPCR, and these were well in agreement with the RNA sequencing outcomes (Fig. S2).

PCA plots, with 95% confidence levels, were created using ClustVis (https://biit.cs.ut.ee/clustvis/), and Venn diagrams were made using http://www.venndiagrams.net/. For age‐related comparisons, enrichment analysis was performed with DAVID version 6.8 (https://david.ncifcrf.gov/gene2gene.jsp), using all genes with *P*_adj_ < 0.1. For dietary comparisons, enrichment analysis of metabolic pathways (of KEGG‐defined pathways) was performed manually using all genes with a *P* < 0.05. Significance of pathway enrichment was determined by the Fisher exact test, using graphpad prism 8.3.0 software package (GraphPad Software, San Diego, CA, USA).

### Statistical analysis

graphpad prism 8.3 software package (GraphPad Software) was used to perform statistical analysis. Data were analysed by 2‐way ANOVA, followed by Tukey's multiple comparisons tests. Correlation graphs were created using a nonparametric Spearman correlation. EE was correlated with body weight, and analysis of EE with body weight as covariate (ANCOVA) was assessed according to the methods described on www.mmpc.org. Data are presented as Tukey's box plots or mean ± SEM, as indicated in the figure legends. Significance is indicated as *P < 0.05, **P < 0.01 and ***P < 0.001.

## Conflict of interest

The authors declare no conflict of interest.

## Author contribution

MBD, MB, JKK and JWJ conceived the study and designed the experiments. MBD, MB and MAVL conducted experiments and performed data analysis. HK, MHK and AG analysed samples. RPvO provided animals. THvD performed the modelling of glucose kinetics. VWB performed data analysis. MBD, MB, MAVL, BMB, JKK and JWJ interpreted the data. MBD, JKK and JWJ wrote the manuscript. JKK and JWJ supervised the study. All authors critically reviewed and edited the manuscript.
