# Supplementary material for: Short‐term protein restriction at advanced age stimulates FGF21 signalling, energy expenditure and browning of white adipose tissue
Source: FEBS J. 2020 Nov 9;288(7):2257–77. doi: 10.1111/febs.15604 (PMC8048886; doi:10.1111/febs.15604)
Supplement: Supplementary file 1 [file FEBS-288-2257-s001.zip › febs15604-sup-0001-Supinfo.pdf]

FEBS PRESS
science publishing by scientists
WILEY
The FEBS Journal
www.febsjournal.org

Short-term protein restriction at advanced age stimulates FGF21 signalling, energy expenditure and browning of white adipose tissue

Marleen B. Dommerholt, Maaike Blankestijn, Marcel A. Vieira-Lara, Theo H. van Dijk, Henk Wolters, Mirjam H. Koster, Albert Gerding, Ronald P. van Os, Vincent W. Bloks, Barbara M. Bakker, Janine K. Kruit and Johan W. Jonker

DOI: 10.1111/febs.15604

Supplementary Material

Supplementary table 1. **Dietary composition.** Diets varying in protein/carbohydrate ratio were used to investigate the metabolic effects in the context of ageing. Diets were based on the AIN-93G breeding diet, obtained from Open Source Diets, containing 7% (low protein), 20% (medium protein) of 40% (high protein).

	D17041406	D17041407	D17041408
	Low Protein (7kcal%)	Med Protein (20kcal%)	High Protein (40kcal%)
Ingredient	gm	gm	gm
Casein	79	220	441
Cysteine	3	3	3
Corn Starch	471	368	203.3
altodextrin 10	156.4	122.2	67.5
Sucrose	107.078	107.078	107.078
Cellulose	50	50	50
Soybean Oil	91	90	88
t-butylhydroquinone	0.014	0.014	0.014
Mineral Mix S10022C	3.5	3.5	3.5
Calcium Carbonate	9.145	12.495	12.495
Potassium Citrate, 1 H_20	1.458	1.773	7.928
Potassium Phosphate, monobasic	8.161	7.763	0
Calcium Phosphate, dibasic	4.55	0	0
Sodium Chloride	2.59	2.59	2.59
Vitamin Mix V10037	10	10	10
Choline Bitartrate	2.5	2.5	2.5
FD&C Yellow Dye #5	0	0.05	0.025
FD&C Red Dye #40	0.05	0	0
FD&C Blue Dye #1	0	0	0.025
Total	999.446	1000.963	998.955
kcal/gm	4.1	4	3.9

Supplementary Figure 1

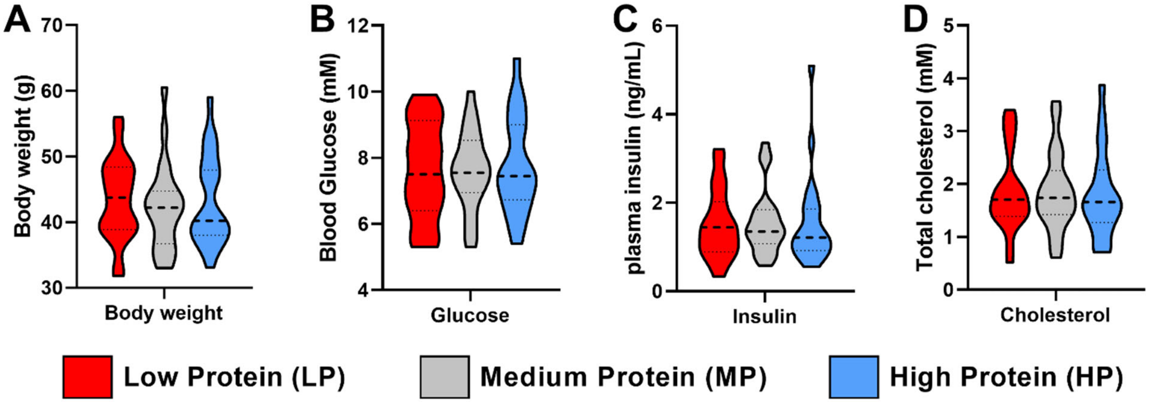

E

	LP			MP			HP		
	Mean	SEM	N	Mean	SEM	N	Mean	SEM	N
BW	43.5	1.32	22	42.3	1.41	22	43.2	1.46	22
Glucose	7.60	0.31	22	7.61	0.25	22	7.75	0.31	22
Insulin	1.52	0.17	22	1.55	0.16	22	1.54	0.22	22
Cholesterol	1.86	0.16	22	1.86	0.16	22	1.80	0.18	22

F

p-value	BW	Glucose	Insulin	Cholesterol
LP vs. MP	0.8183	0.9999	0.9926	0.9997
LP vs. HP	0.9903	0.9351	0.9957	0.9543
MP vs. HP	0.8851	0.9391	0.9996	0.9612

Supplementary figure 1: Normalization of aged animals into experimental groups. Physiological parameters were measured after a 4h fast prior to the experiment, including body weight (A), blood glucose (B), plasma insulin (C) and total plasma cholesterol (D) (n=22). E) Mean, SEM and N of the experimental diets excluding animals who reached humane endpoint. F) p-values, using Ordinary one-way ANOVA between different experimental diets. In all graphs, LP=red, MP=grey, HP=blue.

Supplementary Figure 2

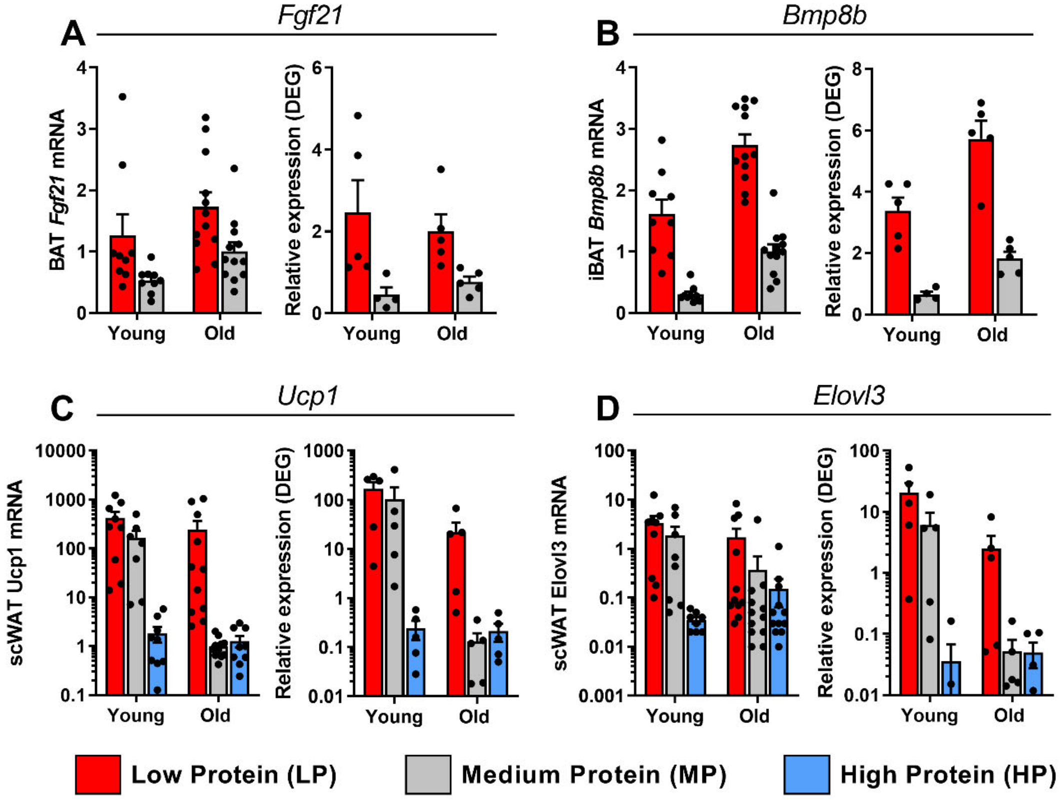

Supplementary figure 2: Validation of RNA sequencing data. Comparing RNA sequencing data (n=4-5) of different genes to qPCR results (young; n=9, old; n=12), including *Fgf21* (A) and *Bmp8b* (B) in brown adipose tissue, and *Ucp1* (C) and *Elovl3* (D) for scWAT. All graphs show mean ± SEM as well as individual samples, LP=red, MP=grey, HP=blue.

Data S1 Supplementary methods

Calculation of Glucose Kinetics

Tracer concentrations were calculated as the product of the blood glucose concentration and the fractional contribution of the tracer at each time point (Suppl. figure 3B, equation 1). To describe changes in blood glucose kinetics in mice, we used the minimal model for glucose metabolism in humans as introduced by Bergman et al. that was further developed for oral glucose tests by Dalla Man and Cobelli et al. (Suppl. figure 3A) [1–6]. We have further modified this model for use in mice by adjusting the restrictions in the total sample volume that can be taken in experiments in mice. To generate sufficient input data, measured data of blood glucose, insulin and tracer concentrations were fitted into equation 2 (Suppl. figure 3B) (SAAM II v2.1, The Epsilon Group, Charlottesville, VA, USA). By interpolations, concentrations of all three metabolites were estimated at multiple time points (t). The bioavailability (F) (Suppl. figure 3B, equation 3) and delay (d) of the bolus were estimated from tracer curves. The model (Minimal Model for Mice, i.e., MiniM) is presented in Figure A. Insulin-mediated, glucose-mediated, and -independent glucose utilization fluxes, peripheral and hepatic insulin sensitivity as well as glucose effectiveness upon administration of a glucose bolus were estimated using the SAAM II software (SAAM II v2.3.1.1, The Epsilon Group, Charlottesville, VA, USA).

For execution of this model, additional parameters were introduced. For flexibility of the model, delay time (d) and bioavailability (F) were introduced as Bayesian values (1.0 ± 0.1) called adjustment parameters A_d and A_F, respectively. Next to this, the volume of the accessible glucose pool, V_g, was adapted from literature on humans [7,8] and the insulin independent glucose utilization under basal conditions (U_{iig}) was set to three times the insulin dependent glucose utilization (U_{idg}) [9,10]. Furthermore, the independent glucose utilization under basal conditions was set to flux at 45% (F_{iig}) of the endogenous glucose production (EGP), rather than an absolute value as was used in humans. In our hands, an absolute value that was valid for all mice strings under very different conditions could not be generated. Above parameters were set as Bayesian values in the model including 10% standard deviation. As result, the fractional turnover rates k_0 to k_4 were estimated as well as the fractional turnover rates determining the utilization fluxes, i.e., independent flux (k_{iic} + k_{idc}), glucose mediated flux (k_{iig}) and insulin mediated flux (k_{idi}).

A

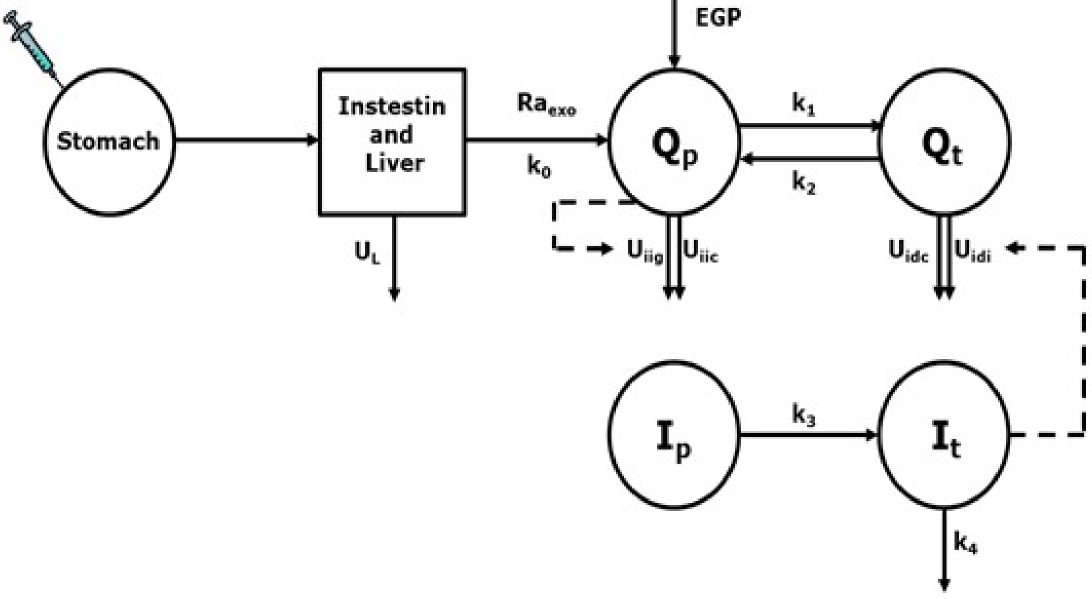

B

Equations for assessing glucose metabolism, using kinetic parameters.

Equation 1:

Calculation of tracer concentrations from blood glucose concentration and the fractional contribution of the tracer at each time point.

$$\left[{}^{13}C_2\text{–glucose}\right]_t = (M_2)_t \cdot [\text{glucose}]_t$$

Equation 2:

Generation of sufficient input, based on measured data of glucose, insulin and tracer concentration.

$$C_t = C_b + C_{(1)_0} \cdot e^{-k_{(1)}(t-d)} + C_{(2)_0} \cdot e^{-k_{(2)}(t-d)} - C_{(3)_0} \cdot e^{-k_{(3)}(t-d)}$$

Equation 3:

Bioavailability of the bolus

$$F = 1 - \left[\frac{C_{(3)_0} k_{(1)} k_{(2)}}{C_{(1)_0} k_{(2)} k_{(3)} + C_{(2)_0} k_{(1)} k_{(3)}}\right]$$

Equation 4:

Glucose clearance rate of basal level

$$GCR_b = V_g * \left[k_{iic} + k_{iig} + \frac{k_{idc} * k_1}{k_{idc} + k_2}\right]$$

Equation 5:

Endogenous glucose production

$$EGP_b = GCR_b * [glc]_b$$

Equation 6:

Insulin action

$$sk = \frac{k_{idc}}{[ins]_b}$$

Equation 7:

Peripheral insulin sensitivity

$$Si = V_g * \frac{k_{idc}}{[ins]_b} * \frac{k_1 * k_2}{(k_{idc} + k_2)^2}$$

Equation 8:

Hepatic insulin sensitivity

$$SHi_t = -\left(\frac{EGP_t - EGP_t}{Ins_t - Ins_b}\right) * \left(\frac{1}{Glc_b}\right)$$

Equation 9:

Hepatic glucose responsiveness

$$SHg_t = -\left(\frac{EGP_t - EGP_t}{glc_t - glc_b}\right) - SHi_t$$

Supplementary Figure 3: Compartmental model for calculating kinetic parameters of glucose metabolism upon an OGTT in mice. A) An oral gavage was given, which passes the liver to contribute to the accessible plasma glucose pool (Q_p) and to the inaccessible “tissue” glucose pool (Q_t). Additionally, glucose is produced/released by the tissues such as the intestine and liver also contribute to Qp. As such, two rates of appearances can be distinguished, namely that of exogenous injected glucose (Ra_{exo}) and of endogenous glucose (EGP). The Qp is in equilibrium with Qt via two rate constants (k_1 and k_2). Disposal of glucose (U) from the model can be divide into insulin-independent glucose-dependent disposal (U_{iig}), insulin-independent constant disposal (U_{iic}), insulin-dependent constant disposal (U_{idc}) and insulin-dependent disposal (U_{idi}). As with glucose, insulin is also distributed over two compartments in this model, namely the accessible plasma insulin pool (I_p) and the inaccessible tissue insulin pool (I_t). B) Insulin action, as well as peripheral and hepatic insulin sensitivity were estimated using varying fluxes and insulin concentrations, in which b indicates basal values measured before the experiment and 0 indicates the estimated value at the time of injection. Abbreviations; k_0-k_4: fractional turnover rates, V_g: volume of the accessible pool, k_{iic}-k_{iig}-k_{idc}: fractional turnover rates determining the utilization fluxes.

References

1. Bergman RN, Ider YZ, Bowden CR, Cobelli C. Quantitative estimation of insulin sensitivity. Am J Physiol Endocrinol Metab Gastrointest Physiol. 1979;5(6).

2. Dalla Man C, Caumo A, Basu R, Rizza R, Toffolo G, Cobelli C. Measurement of selective effect of insulin on glucose disposal from labeled glucose oral test minimal model. Am J Physiol - Endocrinol Metab. 2005;289(5 52-5):909–14.

3. Dalla Man C, Camilleri M, Cobelli C. A system model of oral glucose absorption: Validation on gold standard data. IEEE Trans Biomed Eng. 2006 Dec;53(12):2472–8.

4. Dalla Man C, Toffolo G, Basu R, Rizza RA, Cobelli C. A model of glucose production during a meal. In: Annual International Conference of the IEEE Engineering in Medicine and Biology - Proceedings. 2006. p. 5647–50.

5. Dalla Man C, Piccinini F, Basu R, Basu A, Rizza RA, Cobelli C. Modeling hepatic insulin sensitivity during a meal: Validation against the euglycemic hyperinsulinemic clamp. Am J Physiol - Endocrinol Metab. 2013 Apr 15;304(8):E819-25.

6. Cobelli C, Man CD, Toffolo G, Basu R, Vella A, Rizza R. The oral minimal model method. Diabetes. 2014 Apr;63(4):1203–13.

7. Tissot S, Normand S, Guilluy R, Pachiaudi C, Beylot M, Laville M, et al. Use of a new gas chromatograph isotope ratio mass spectrometer to trace exogenous 13C labelled glucose at a very low level of enrichment in man. Diabetologia. 1990 Aug;33(8):449–56.

8. Gastaldelli A, Coggan AR, Wolfe RR. Assessment of methods for improving tracer estimation of non-steady- state rate of appearance. J Appl Physiol. 1999 Nov;87(5):1813–22.

9. Caumo A, Cobelli C. Hepatic glucose production during the labeled IVGTT: estimation by deconvolution with a new minimal model. Am J Physiol. 1993 May;264(5 Pt 1):E829-41.

10. Gottesman I, Mandarino L, Gerich J. Estimation and kinetic analysis of insulin-independent glucose uptake in human subjects. Am J Physiol - Endocrinol Metab. 1983;7(6).

Supplementary table 2: **Sybr Green primer sequence**

Gene	Description	Forward primer (5' to 3')	Reverse primer (5' to 3')
36B4	acidic ribosomal phosphoprotein	GCT CCA AGC AGA TGC AGC A	CCG GAT GTG AGG CAG CAG
Bmp8b	Bone Morphogenetic Protein 8b	TCA ACA CAA CCC TCC ACA TCA	AGA TCG GAG CGT CTG AAG ATC
Cd137	Tumor Necrosis Factor Receptor Superfamily Member 9	CGT GCA GAA CTC CTG TGA TAA C	GTC CAC CTA TGC TGG AGA AGG
Cidea	Cell Death-Inducing DFFA-Like Effector A	GGC CGT GTT AAG GAA TCT GC	GTA TGT GCC CGC ATA GAC CA
Elovl3	Elongation Of Very Long Chain Fatty Acids Protein 3	TCC ATG AAT TTC TCA CGC GG	GCT TAC CCA GTA CTC CTC CAA
Ki67	antigen identified by monoclonal antibody Ki 67	TCA CCT GGT CAC CAT CAA GC	TCA ATA CTC CTT CCA AAC AGG CA
Prdm16	PR Domain Containing 16	CAA GGA GGA GGA GAG AGA TTC C	AAC GTC ACC GTC ACT TTT GG
Tmem26	transmembrane protein 26	GGA CGA GGC TAC AAA TGG CT	GGT GCT GCA ATA CTG GTT TCC
Ucp1	uncoupling protein 1	CAC GGG GAC CTA CAA TGC TT	TAG GGG TCG TCC CTT TCC AA

Supplementary table 3: **Taqman primer sequence**

	Description	Forward primer (5' to 3')	Reverse primer (5' to 3')	Probe (5' to 3')
36B4	acidic ribosomal phosphoprotein	GCT TCA TTG TGG GAG CAG ACA	CAT GGT GTT CTT GCC CAT CAG	TCC AAG CAG ATG CAG CAG ATC CGC
Fgf21	fibroblast growth factor 21	CCG CAG TCC AGA AAG TCT CC	TGA CAC CCA GGA TTT GAA TGA C	CCT GGC TTC AAG GCT TTG AGC TCC A
Ppargc1a	peroxisome proliferative activated receptor,gamma,coactivator 1	GAC CCC AGA GTC ACC AAA TGA	GGC CTG CAG TTC CAG AGA GT	CCC CAT TTG AGA ACA AGA CTA TTG AGC GAA CC
